# Selective Functionalization of Arene C(sp^2^)–H Bonds by Gold Catalysis: The Role of Carbene Substituents

**DOI:** 10.1021/acscatal.2c01713

**Published:** 2022-05-25

**Authors:** Juan Diego Pizarro, Inga L. Schmidtke, Ainara Nova, Manuel R. Fructos, Pedro J. Pérez

**Affiliations:** § Laboratorio de Catálisis Homogénea, Unidad Asociada al CSIC, CIQSO-Centro de Investigación en Química Sostenible and Departamento de Química, Universidad de Huelva, 21007 Huelva, Spain; † Department of Chemistry, Hylleraas Centre for Quantum Molecular Sciences and Centre for Materials Science and Nanotechnology, University of Oslo, N-0315 Oslo, Norway

**Keywords:** gold catalysis, gold-carbenes, carbene transfer, Profen skeletons, DFT studies

## Abstract

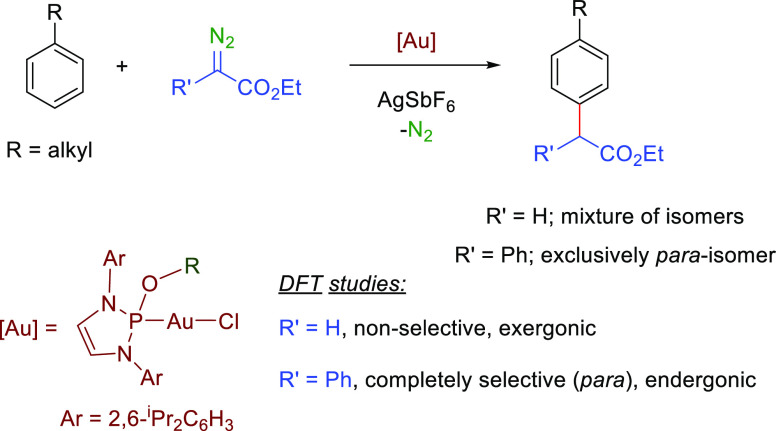

The complete regioselective incorporation of carbene units to nonactivated arene rings has been achieved employing gold(I) catalysts bearing alkoxydiaminophosphine ligands, with readily available, nonelaborated ethyl 2-phenyldiazoacetate as the carbene source. These results are in contrast with the scarce precedents which required highly elaborated diazo substrates. Density functional theory (DFT) calculations have revealed the important role of the R group in the C(R)CO_2_Et fragment, which dramatically affects the energy profile of this transformation.

Gold catalysis has emerged in the current century as an important tool in the area of carbon–hydrogen bond functionalization reactions.^1^ The formation of gold-carbene species upon direct activation of triple carbon–carbon bonds provided a number of transformations involving such C–H bond modification.^2^ At variance with that, it was not until 2005 that the first example of a gold-catalyzed carbene transfer reaction from a diazo compound was reported.^3^ With benzene as the model substrate, the formal insertion into the aromatic C–H bond was observed (Scheme 1a). It is worth mentioning that we employ herein the term insertion albeit mechanistic studies^4,5^ have shown that this is not the true pathway, contrary to the C(sp^3^)–H bond functionalization where the metal-carbene inserts into such a moiety.^6^ Since then, many examples employing that strategy have been reported,^7^ allowing the neat functionalization of aliphatic or aromatic carbon–hydrogen bonds. Regarding the latter, the use of monosubstituted benzenes as substrates has attracted some attention, in view of its potential synthetic use, for which gold seems to be the metal of choice. The nature of the substituent at the benzene ring greatly influences the catalytic reaction. Liu and Zhang employed^8^ gold-based catalysts for the functionalization of activated rings such as phenol and *N*-acylanilines, observing complete incorporation of the carbene in the *para* position with the OH group remaining unreacted (Scheme 1b). Lan and Shi later reported^9^ on the modification of anisole and anilines in a similar manner. In both cases, phosphite-derived ligands accompanied gold in the catalyst precursors. On the other hand, for monosubstituted benzenes lacking those activating groups, the selective functionalization is not so favored. Only the smart use of modified donor–acceptor diazo compounds,^10^ either introducing a CF_3_ moiety in the ester side or halide/CF_3_ substituents in the aromatic ring of the diazo compound, led Liu and Zhang^11^ to reach significantly high regioselectivities (Scheme 1c).

**Scheme 1:**
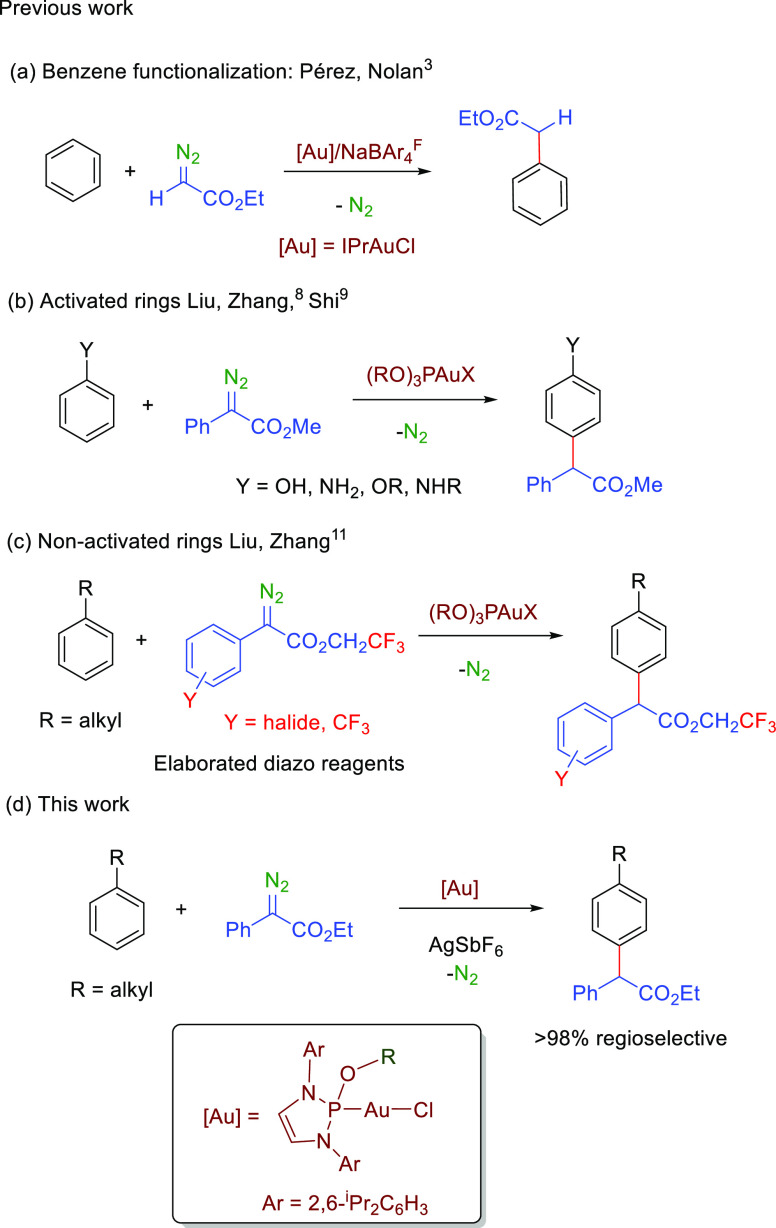
Gold-Catalyzed Direct Arene Functionalization by Carbene Transfer from Diazo Compounds

In this contribution, we report the catalytic properties of a family of gold complexes containing alkoxydiaminophosphine ligands (ADAP)^12^ which promote the exclusive incorporation of carbene units into the C–H bond located at the *para* position of monosubstituted alkylbenzenes with the readily available ethyl 2-phenyldiazoacetate as the carbene source (Scheme 1d), not needing the elaboration on the diazo reagent. Computational studies have revealed the crucial role of the arene group in the selectivity, explaining the large differences in comparison with the widely employed ethyl diazoacetate reagent.^13^

We have recently described^12^ the synthesis and characterization of a series of compounds of general formula (ADAP)AuCl, bearing alkoxydiaminophosphine ligands containing a five-membered ring that resembles that of NHC ligands. In view of our previous work with (NHC)AuCl compounds as precatalysts for carbene transfer reactions to C(sp^3^)–H and C(sp^2^)–H bonds^3,14,15^ and the above-mentioned success^8,9,11^ of phosphite-containing gold catalysts for selective C(sp^2^)–H bond functionalization, we decided to test their catalytic properties toward this transformation. In a first screening, complex **1a** (Table 1) was used as a catalyst precursor in the reaction of toluene as the model substrate and two representative diazo compounds, ethyl diazoacetate (EDA) and ethyl 2-phenyldiazoacetate (PhEDA), with AgSbF_6_ as the halide scavenger. The array of experiments was carried out with applying a 1:20:2000 ratio of catalyst:diazo:toluene. In this transformation, the chemoselectivity refers to the potential formation of four different types of compounds (Table 1),^5^ originating from (i) the insertion of the carbene into the methyl C(sp^3^)–H bond; (ii) the related modification of the C(sp^2^)–H bonds; (iii) the Buchner reaction, leading to cycloheptatrienes, and (iv) the coupling of two carbene units, accounting for a total of nine different compounds. The results shown in Table 1 indicate that the gold-based catalyst does not induce modification at the methyl substituent (entries 1–4) and that the Buchner reaction is only observed with EDA (entries 1 and 2). Regarding the functionalization of the C(sp^2^)–H bonds, both diazo compounds provide products derived from the formal insertion of the carbene group into such bonds; however, whereas EDA gives a mixture of the three *o*-, *m*-, and *p*-isomers, only the *para* isomer (**2**-**p**) is observed with PhEDA as the carbene precursor under the applied reaction conditions. The conditions employed resulted from the optimization of the different variables (see the SI). The use of a donor–donor diazocompound such as diphenyldiazomethane gave no arene-functionalized products under the same conditions; most of the diazo remained unaltered, with some tetraphenylethylene being formed from the carbene coupling reaction.

**Table 1:**
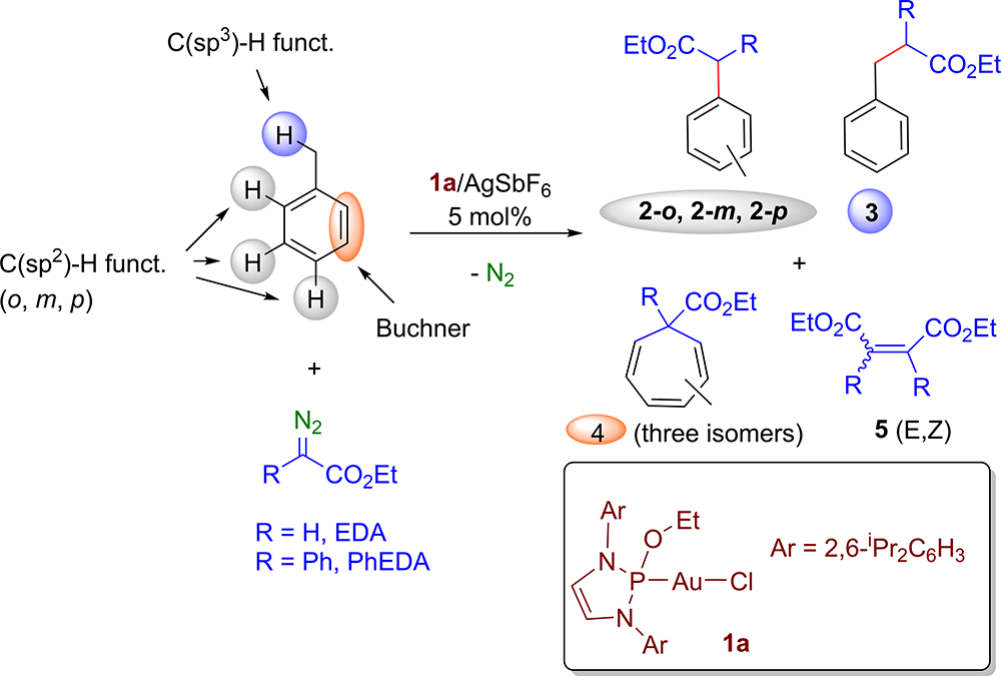
Functionalization of Toluene by Carbene Transfer Using Complex **1a** as a Precatalyst^a^

entry	R	yield % **2/3/4/5**	regioselectivity to *o*:*m*:*p* in compds **2**
1	H^b^	64/nd/32/4	41/20/39
2	H^c^	68/nd/28/4	40/26/34
3	Ph^b^	12/nd/nd/88	nd/nd/>98
4	Ph^c^	67/nd/nd/33	nd/nd/>98

a Reaction conditions: diazo compound (0.25 mmol), toluene (25 mmol), **1a** (5 mol % referred to diazo compound), AgSbF_6_ (5 mol % referred to diazo compound), DCM (5 mL). Yields measured by ^1^H NMR spectroscopy using benzaldehyde as the internal standard. See the Supporting Information for complete optimization details.

b Diazo added in one portion.

c Diazo added in seven portions, one portion every 30 min.

The results obtained with **1a** prompted us to evaluate the series of gold complexes **1b**–**f** (Scheme 2) as precatalysts for the reaction of toluene and PhEDA, with the excellent finding that all of them gave only one toluene-derived product, that of the functionalization in the *para* position relative to the methyl group of toluene. Chemoselectivity was affected by the catalyst precursor, since the yield of the functionalization product **2-p** varied from 63 to 67% for **1a**–**1d** to 73% with **1e** and 86% with **1f**, with the remaining initial diazo compound being converted into olefins **5**.

**Scheme 2:**
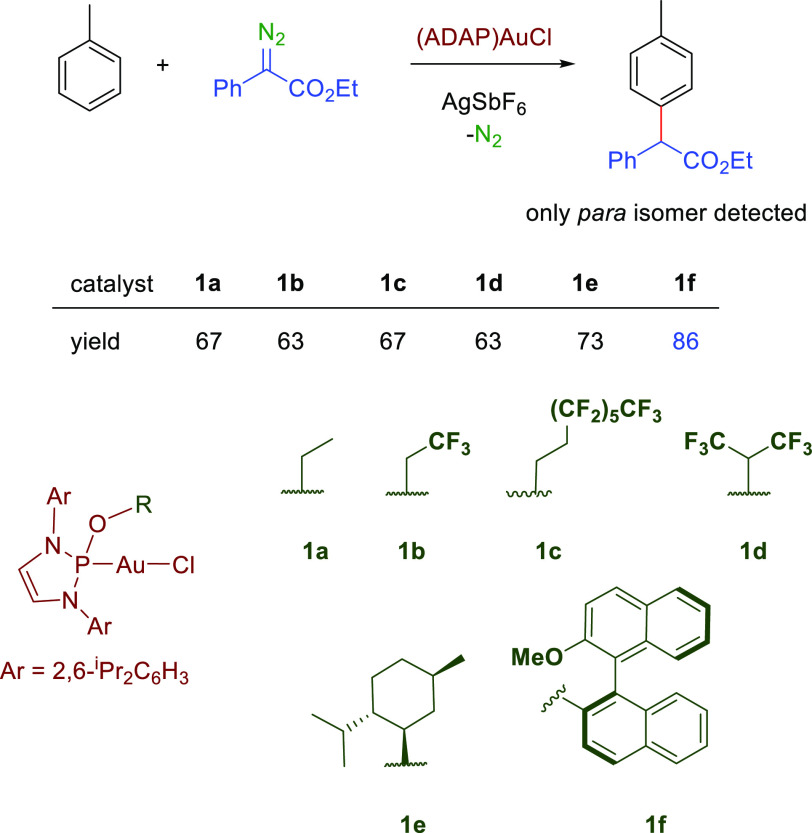
Catalyst Screening for Toluene Functionalization Reaction conditions are the same as those noted in Table 1.

The use of other alkylbenzenes as substrates showed the same excellent regioselectivity toward the *para* isomer (Scheme 3a). Ethyl- and isobutylbenzenes were studied as representative examples, for which compounds **6** and **7** were obtained as the unique arene derivatives in 83% and 78% isolated yields. Carbene-dimers **5** accounted for all initial PhEDA. To complete this study, the electron-rich arenes phenol, anisole, and dimethylaminobenzene were tested, with the *para* isomers **8**, **9**, and **10** being obtained, respectively, in nearly quantitative yields. Our gold catalyst **1f** shows complete selectivity toward C(sp^2^)–H bonds, despite the presence of C(sp^3^)–H bonds and employing nonelaborated PhEDA. Notably, this is achieved without the need of introducing electron-withdrawing groups in the diazo reagents.^8,9,11^ For the sake of comparison, we also tested those more elaborated diazo reagents, employing isobutylbenzene as the substrate (Scheme 3b), given its importance as a Profen skeleton. Three diazo compounds bearing a CF_3_ group at the ester end and either H, Br, or CF_3_ at the aryl end were prepared and used in these experiments. As shown in Scheme 3b, the exclusive formation of the *para* isomer was maintained. This illustrates how the modified diazo reagents combined with our catalyst only affect the chemoselectivity, with yields for **11**–**13** being in the 91–97% interval. Interestingly, the chiral nature of complex **1f** does not induce any enantiomeric excess in the corresponding products. Previous work^4,5^ has proposed the formation of Wheland-like intermediates (Scheme 4) and the participation of enol species which afford the final products in a water-assisted process. In good accord with this, when D_2_O was added to our reaction mixtures, partial deuteration was observed at the −C(H/D)(Ph)(CO_2_Et) fragment.

**Scheme 3:**
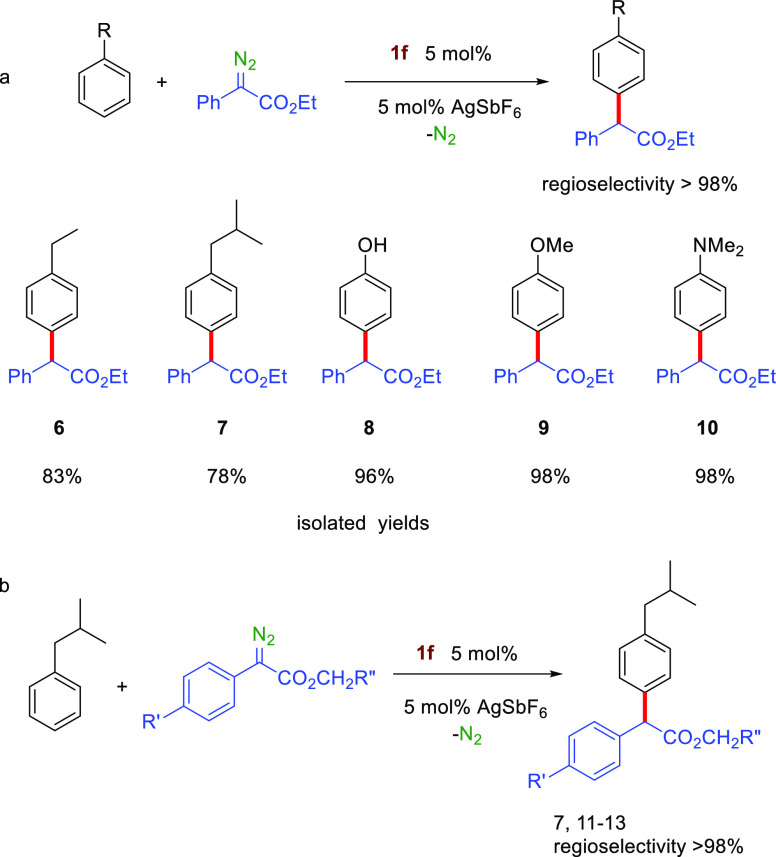
Monosubstituted Benzene Catalytic Functionalization: (a) Effect of the Substituent and (b) Effect of the Diazo Compound Reactions carried out under the standard conditions shown in Table 1.

**Scheme 4:**
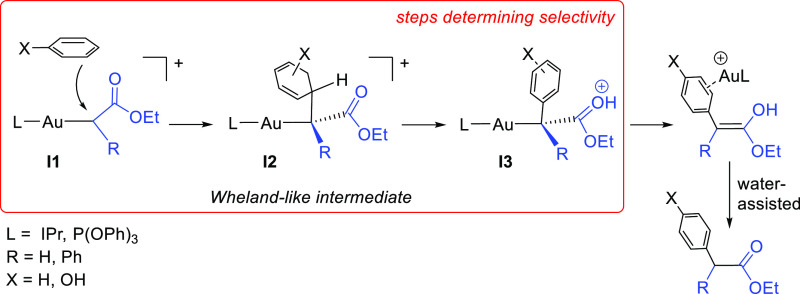
Reported Mechanism^4,5^ for Arene Functionalization by Gold-Carbene Complexes

The combination of the gold-ADAP complexes and PhEDA provides an unprecedented regioselectivity for such a low-elaborated diazo reagent. Reported mechanistic work^4,5^ has shown that the reaction takes place by an electrophilic attack of the gold-carbene species onto the arene ring. However, those examples correspond either to EDA as the carbene source and benzene as the substrate^5^ or to PhEDA as the diazo compound and the electron-rich phenol as the substrate.^4^ In this work, we have used density functional theory (DFT) calculations (PBE0-D3/def2SVP//def2TZVP with SMD solvation) to account for the larger regioselectivity observed with PhEDA compared to EDA under the same conditions, with our gold-ADAP complex **1a** and toluene as the model substrate.

The energy profile of the addition of toluene to the gold-carbene **I1a**, followed by proton migration to the carbonyl group of the ester, is shown in Figure 1. Other pathways involving different orientations of the phosphine ligand and the ester group on the active catalyst were also considered but showed higher barriers. The same applies for the methyl group of toluene in the *ortho* C–H bond activation step (see SI). The overall reaction of EDA with toluene is strongly exergonic with relatively low energy barriers in the addition step, which barely differ for the *para* and *ortho* isomers (6.3 and 6.9 kcal mol^–1^, respectively). These values explain the low selectivity found with such a diazo reagent (Table 1, entries 1 and 2). Contrary, when PhEDA serves as the carbene source, an endergonic process is found for the formation of the **I2a-Ph** regioisomers. Additionally, a stronger dependency of the energies on the orientation of the methyl group of toluene was observed for this system, resulting in energy barriers differing by around 3 kcal mol^–1^ (14.3 kcal mol^–1^ and 17.5 kcal mol^–1^ for the *para* and *ortho* isomers, respectively). This change in dependency is also apparent in the C–C bond formation distances of **TSI1a-I2a-H** and **TSI1a-I2a-Ph**, which are *ca*. 1 Å shorter in the PhEDA system (2.103 and 2.187 Å for the *para* and *ortho* additions, respectively, compared to 3.240 and 3.157 Å with EDA, see the SI). The shorter distance between the substrate and the gold-complex with PhEDA probably accounts for the larger energy difference for the *para* and *ortho* addition TSs, which is consistent with the higher regioselectivity experimentally observed for reactions on PhEDA (Table 1, entries 3 and 4). In both cases, the deprotonation of toluene in **I2a** and subsequent formation of the **I3a** isomers is an exergonic reaction with an estimated barrier of *ca*. 1 kcal mol^–1^ (see the SI). This trend is more pronounced for the EDA system. It is worth noting that the *para* isomer is not only favored kinetically but also thermodynamically when PhEDA is employed as the carbene source. The lower energy barriers obtained with EDA can also explain the minor formation of product **5**, which requires gold-carbene accumulation. While this is more likely with PhEDA due to higher energy barriers, dimerization could be minimized by using bulkier ligands such as **1e** or **1f**.

**Figure 1:**
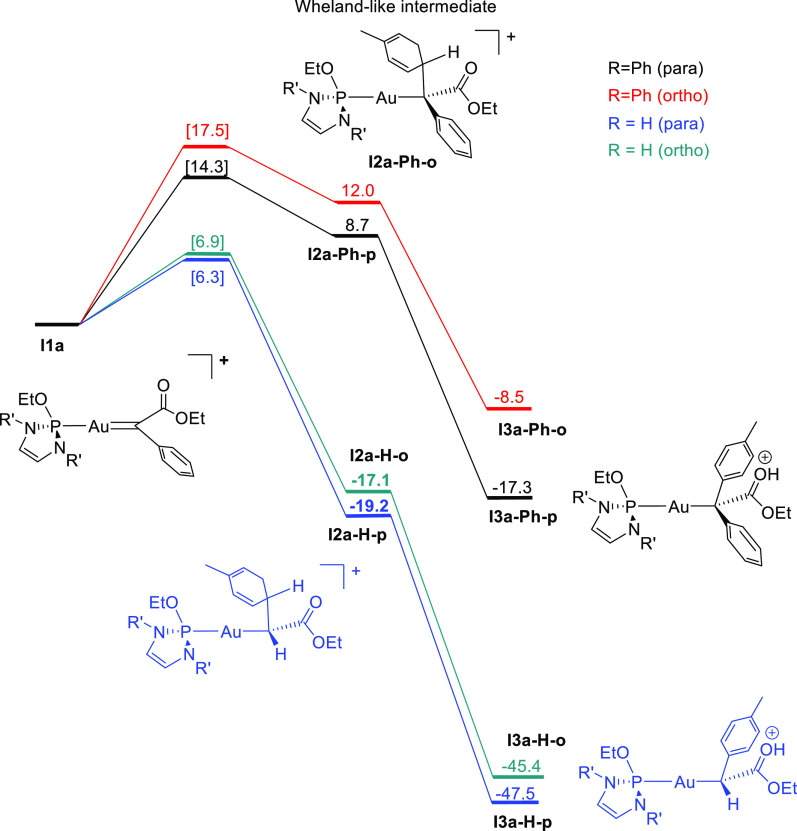
Free energy profiles (in kcal mol^–1^) for the steps defining regioselectivity in the functionalization of toluene with **I1a**-**Ph** and **I1a**-**H** in *para* and *ortho* positions. Transition state energies are shown in brackets.

The energy profile in Figure 1 suggests that the toluene functionalization would be enantioselective when using chiral ligands such as in **1f**. However, the following water assisted keto–enol equilibrium, as shown in Scheme 4, leads to a racemic mixture. The aforementioned experiment with added D_2_O demonstrates such proposal.

This study clearly shows the influence that the carbene R group (R = Ph or H, Table 1) has on the energies for the electrophilic addition, the crucial step accounting for the regioselectivity. To gain more insight, we analyzed the structures of the intermediates **I1a-H** and **I1a-Ph**. For complex **I1a-H**, a Au–C1 bond distance typical for carbene-like gold(I) compounds was found (1.99 Å, Figure 2, see the SI for details).^16^ Despite a slight elongation of the corresponding bond in **I1a-Ph** (2.03 Å), this complex can also be categorized as a carbene complex but with a predominant carbocation-like conformation. It is to be noted that the orientation of the phenyl fragment in the structure of **I1a-Ph** is parallel to the carbene plane, and the bond between the carbenic carbon and the C_*ipso*_(Ph) carbon appears contracted (1.41 Å).^16^ In the structure of **I1a-H**, a narrow angle (α) of 102° between C1 and the carbonyl group was observed as an outstanding feature. In both **I1a-Ph** and **I1a-H** structures, the C=O group is perpendicular to the Au–C–R plane with R–C–C=O dihedral angles of 99.1° for **I1a-Ph** and 81.2° for **I1a-H**.

**Figure 2:**
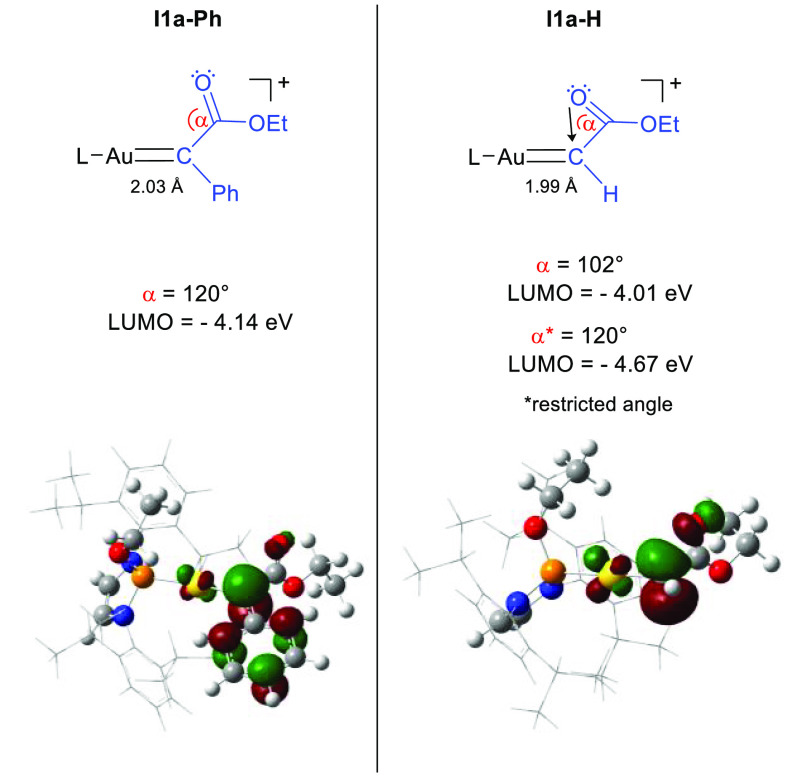
Schematic representation for the LUMO orbital involved in the electrophilic attack for **I1a-Ph** and **I1a-H**, with their corresponding energies for the unrestricted geometries and restricted (**α** = 120^◦^) in the case of **I1a-H** (in eV). Isovalue: 0.05.

Further analysis of **I1a** using natural bond orbitals (NBOs) showed that the main contribution for the stabilization of the carbene center in **I1a-Ph** is derived from the phenyl substituent, observable in the electron donation of the lone-pair on C_*ipso*_(Ph) toward C1 (101.7 kcal mol^–1^, see the SI for details). This strong donation is also reflected in the energy barrier for the rotation of the C1–C_*ipso*_(Ph) bond (21.2 kcal mol^–1^). Since **I1a-H** does not bear such an electron-rich substituent, its main source of stabilization is the π-back-donation from the metal center (from the Au *d* orbital to the C1 *p* orbital; 28.6 kcal mol^–1^). Additionally, an unusual σ-donation from the lone pair of the carbonyl group into the low vacancy orbital of **I1a-H** was observed, which is responsible for the contraction of the CCO angle **α** from 120° in **I1a-Ph** to 102° in **I1a-H** (Figure 2). Despite this interaction not being mentioned in previous computational studies involving EDA,^5,17^ related optimized structures do present a CCO angle smaller than 120° (107°).

The obtained energy of the LUMO computed for **I1a-Ph** (−4.14 eV) was unexpectedly lower than that of **I1a-H** (−3.98 eV), suggesting a larger electrophilicity of **I1a-Ph** and, apparently, contradicting the reactivity trends, which are known to be dominated by the LUMO.^16^ When modeling **I1a-H** with an angle **α** frozen at 120°, the energy of the LUMO decreases to −4.67 eV without changing the potential energy of the intermediate significantly (ΔΔ*E* = 2.6 kcal/mol). The lower energy of the LUMO for I1a-H*, easily reached from **I1a-H**, compared to the LUMO for **I1a-Ph** is consistent with the highest reactivity (lower energy barrier) of the **I1a-H** carbene toward electrophilic addition.

From the above data, we can extract the following: (a) depending on the degree of stabilization of the carbenic carbon through electron donation, the addition of the arene to this carbon is either exergonic (EDA) or endergonic (PhEDA); (b) the orientation of the arene relative to the carbene ligand has a larger influence in the transition state energies for the PhEDA system due to the closest interaction with the Au-complex, which allows differentiation between *ortho* and *para* C–H bonds; (c) both Au=C(R)CO_2_Et intermediates seem to be carbene-like species, albeit that with R = Ph displays a larger contribution of the carbocation resonance form.

In conclusion, we have found that gold complexes bearing alkoxydiaminophosphine (ADAP) ligands promote the completely selective functionalization of monoalkylbenzenes at *para* positions by the formal insertion of carbene groups from the nonelaborated ethyl 2-phenyldiazoacetate. The presence of the aryl ring in the carbene moiety strongly influences the reaction outcome, and its intrinsic effect has been revealed by DFT studies. These findings pave the way to the development of new families of catalysts based on the understanding of the nature of this transformation.

## Supporting Information Available

General methods, synthetic procedures, and catalytic experiments (PDF)
xyz file (XYZ)

## Supplementary Material

cs2c01713_si_001.pdf

cs2c01713_si_002.xyz

## Dedication

Dedicated to Professor Joan Bosch on occasion of his 75th birthday.
