# Supplementary material for: Selective Functionalization of Arene C(sp2)–H Bonds by Gold Catalysis: The Role of Carbene Substituents
Source: ACS Catal. 2022 May 25;12(12):6851–6. doi: 10.1021/acscatal.2c01713 (PMC9210454; doi:10.1021/acscatal.2c01713)
Supplement: Supplementary file 1 — cs2c01713_si_001.pdf [file cs2c01713_si_001.pdf]

Selective functionalization of arene C(sp²)-H bonds by gold catalysis: the role of carbene substituents

Juan Diego Pizarro,[§] Inga L. Schmidtke,[†] Ainara Nova,[*,†] Manuel R. Fructos,[*,§] and Pedro J. Pérez[*,§]

Laboratorio de Catálisis Homogénea, Unidad Asociada al CSIC. CIQSO-Centro de Investigación en Química Sostenible and Departamento de Química. Universidad de Huelva, 21007 Huelva, Spain

Department of Chemistry, Hylleraas Centre for Quantum Molecular Sciences and Centre for Materials Science and Nanotechnology, University of Oslo, N-0315 Oslo, Norway.

perez@dqcm.uhu.es; manuel.romero@dqcm.uhu.es; a.n.flores@kjemi.uio.no.

Table of Contents

1	General Methods	S2
2	General Catalytic Procedure	S2
2.1	Initial studies employing EDA and PhEDA	S2
2.2	Halide scavenger screening and optimization of toluene equivalents.	S3
2.3	Catalyst screening.	S4
2.4	Mono-substituted benzenes catalytic functionalization. Effect of the substituent in the aryl ring.	S5
2.5	Mono-substituted benzenes catalytic functionalization. Effect of the diazo compound	S5
3	Characterization of Products	S6
4	NMR Spectra	S9
5	References experimental	S20
6	Computational details	S21

Experimental Procedures

1. General Methods.

All reactions and manipulations were carried out under a nitrogen atmosphere by using standard Schlenk techniques or under nitrogen atmosphere in an Mbraun glovebox. All substrates were purchased from Aldrich and used without further purification, diazo compounds[1] were prepared according to literature methods. Solvents were distilled and degassed before use. Complexes **1a-1f** were synthetized according to literature procedures.[2,3] NMR spectra were recorded on Agilent 400 MR or Agilent 500 DD2. FTIR spectra were recorded on a Nicolet IR200 FTIR spectrometer. ^{1}H and ^{13}C NMR shifts were measured relative to deuterated solvents peaks but are reported relative to tetramethylsilane. Elemental analyses were performed on a PerkinElmer Series II CHNS/O Analyzer 2400. High Resolution Mass Spectroscopy (HRMS) experiments were carried out at the Centre of Research Technology and Innovation of the University of Seville (CITIUS).

2. General Catalytic Procedure.

2.1. Initial studies employing EDA and PhEDA.

The gold complex (0.0125 mmol), $AgSbF_6$ (0.0125 mmol), toluene (25 mmol) and 5 mL of DCM were added to a Schlenk flask. The diazo compound (0.25 mmol) was added. The resulting mixture was stirred at room temperature for 12 h. The crude was analyzed by ^{1}H NMR and quantified employing benzaldehyde as internal standard. Characteristic ^{1}H NMR signals for the mixture of o-, *m*-, *p*- activation products can be consulted in the literature.[4,5] Products were purified by flash chromatography on silica gel gradient 100 Petroleum ether to 95:5 Petroleum ether:EtOAc.

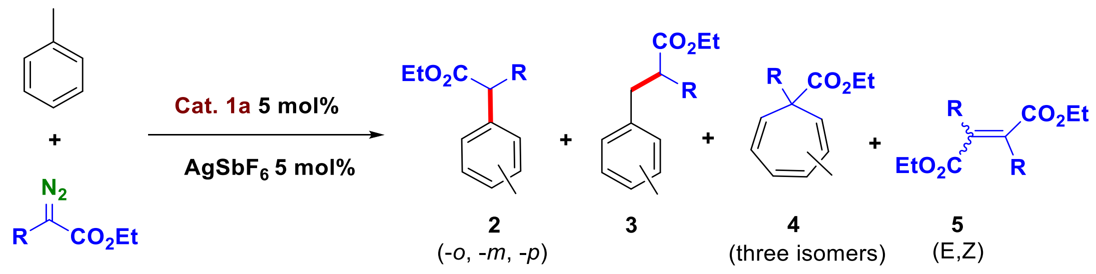

Table S1. Functionalization of toluene by carbene transfer using complex **1a** as precatalyst.[a]

Entry	*R*	*Yield %* *2/3/4/5*	*Regioselectivity to o:m:p in compounds 2*
1	H[b]	64/nd/32/4	41/20/39
2	H[c]	68/nd/28/4	40/26/34
3	Ph[b]	12/nd/nd/88	nd / nd />98
4	Ph[c]	67/nd/nd/33	nd / nd />98

[a]Reaction conditions: diazo compound (0.25 mmol), toluene (25 mmol), [1a] (5 mol% referred to diazo compound), AgSbF6 (5 mol% referred to diazo compound), DCM (5 mL). Yields measured by ^{1}H NMR spectroscopy using benzaldehyde as internal standard. [b]Diazo added in one portion. [c]Diazo added in seven portions, one portion every 30 min.

2.2. Halide scavenger screening and Optimization of toluene equivalents.

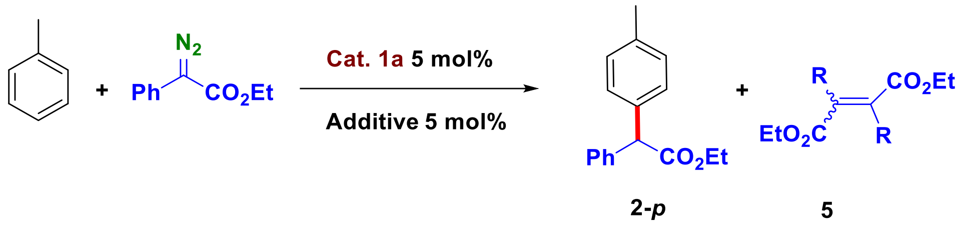

Table S2. Halide scavenger screening using complex **1a** as precatalyst.[a]

Entry	*Additive*	*Yield (%)* ***2-p***	*Yield (%)* ***5***
1	$AgSbF_6$	67	33
2	$AgPF_6$	58	42
3	$AgBF_4$	54	46
4	$NaBAr^F_4$	51	49
5	$AgNTf_2$	-	-

[a]Reaction conditions: diazo compound (0.25 mmol), toluene (25 mmol), [1a] (5 mol% referred to diazo compound), additive (5 mol% referred to diazo compound), DCM (5 mL). Diazo added in seven portions, one portion every 30 min. Yields measured by ^{1}H NMR spectroscopy using benzaldehyde as internal standard.

The isolated ionic complex [(ADAP)Au(NCMe)]SbF_6 was employed as the catalyst, the reaction outcome being identical to that obtained from **1a**+$AgSbF_6$. At variance with that, isolated (ADAP)Au(NTf_2) did not induce the catalytic transformation.

Table S3. Optimization of toluene equivalents.

Entry	*Equiv.*	*Conversion (%)*	*C-H Func.(%)* **2** *(-o/-m/-p)*	*Yields (%)*
1	10	>99	nd/nd/>98	26
2	20	>99	nd/nd/>98	30
3	50	>99	nd/nd/>98	46
4	100	>99	nd/nd/>98	67
5	200	>99	nd/nd/>98	67

Reaction conditions as those noted in Table S2. The remaining initial PhEDA (up to 100%) was converted into dimers.

2.3. Catalyst screening.

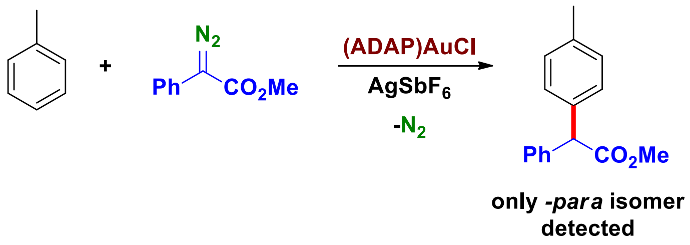

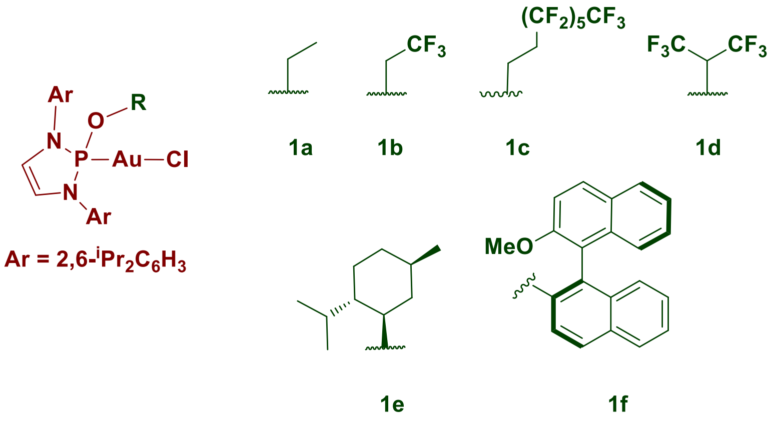

Table S4. Catalyst screening.

Entry	*Cat.*	*Yield (%)*
1	**1a**	67
2	**1b**	63
3	**1c**	67
4	**1d**	63
5	**1e**	73
6	**1f**	86

Reaction conditions as those noted in Table S2.

2.4. Mono-substituted benzenes catalytic functionalization. Effect of the substituent in the aryl ring.

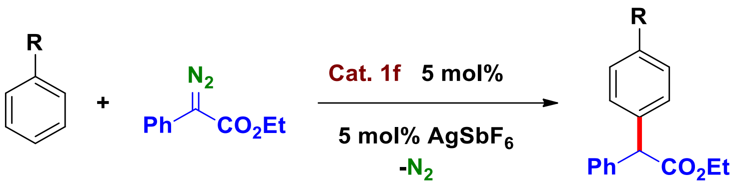

	6	7	8	9	10
R	Et	Isobutyl	OH	OMe	NMe_2

Table S5. Effect of the substituent.

Entry	*R*	*Isolated Yield (%)*
1	Et	83
2	Isobutyl	78
3	OH[a]	96
4	OMe[a]	98
5	NMe_2[a]	98

Reaction conditions as those noted in Table S2. [a] 10 equiv. of substrate was employed.

2.5. Mono-substituted benzenes catalytic functionalization. Effect of the diazo compound.

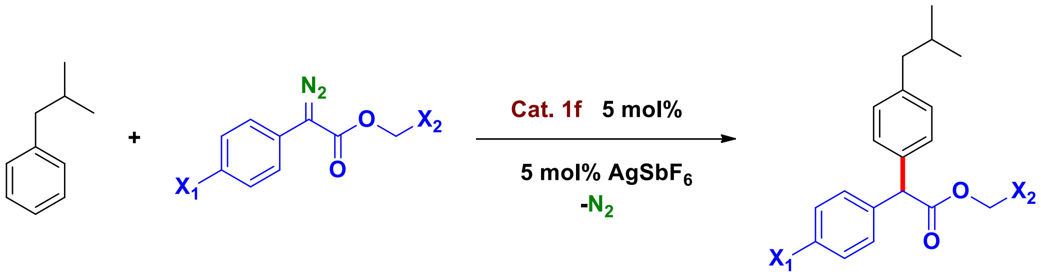

	7	11	12	13
X_1	H	H	Br	CF_3
X_2	CH_3	CF_3	CF_3	CF_3

Table S6. Effect of the diazo compound.

Entry	*X_1/X_2*	*Isolated Yield (%)*
1	H/CH_3	78
2	H/CF_3	91
3	Br/CF_3	95
4	CF_3/CF_3	97

Reaction conditions as those noted in Table S2. The remaining initial diazocompound (up to 100%) was converted into dimers.

3. Characterization of Products.

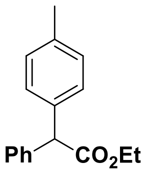

Ethyl 2-phenyl-2-(p-tolyl)acetate **2** (54 mg, 86% yield). **^{1}H NMR** (400 MHz, $CDCl_3$): δ 7.35 (d, 4H, CH_{Ar}), 7.30-7.22 (m, 3H, CH_{Ar}), 7.16 (d, 2H, CH_{Ar}), 5.01 (s, 1H, Ph*CH*CO_2Et), 4.23 (q, 2H, *J* = 7.1 Hz, $CO_2CH_2CH_3$), 2.35 (s, 3H, CH_3Ph), 1.28 (t, 3H, *J* = 7.1 Hz, $CO_2CH_2CH_3$). **^{13}C{^{1}H} NMR** (100 MHz, $CDCl_3$): δ 172.6 (*C*=O), 139.0 (C_{Ar}), 136.8 (C_{Ar}), 135.8 (C_{Ar}), 129.3 (C_{Ar}), 128.5 (C_{Ar}), 128.4 (C_{Ar}), 127.1 (C_{Ar}), 61.1 ($CO_2CH_2CH_3$), 56.7 (Ph*C*HCO_2Et), 21.0 (CH_3Ph), 14.1 ($CO_2CH_2CH_3$). **HMRS (ESI)**: Mass calculated for $C_{17}H_{18}O_2Na$ $[M+Na]^+$, 277.1204. Found $[M+Na]^+$, 277.1199.

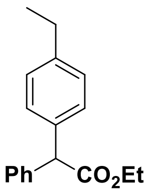

Ethyl 2-(4-ethylphenyl)-2-phenylacetate **6** (56 mg, 83% yield). **^{1}H NMR** (400 MHz, $CDCl_3$): δ 7.39-7.27 (m, 4H, CH_{Ar}), 7.30-7.21 (m, 3H, CH_{Ar}), 7.17 (d, 2H, CH_{Ar}), 4.98 (s, 1H, Ph*CH*CO_2Et), 4.26 (q, 2H, *J* = 7.1 Hz, $CO_2CH_2CH_3$), 2.62 (q, 2H, *J* = 7.0 Hz, PhCH_2CH_3), 1.27 (t, 3H, *J* = 7.1 Hz, $CO_2CH_2CH_3$), 1.21 (t, 3H, *J* = 7.0 Hz, PhCH_2CH_3). **^{13}C{^{1}H} NMR** (100 MHz, $CDCl_3$): δ 172.7 (*C*=O), 143.1 (C_{Ar}), 138.9 (C_{Ar}), 136.0 (C_{Ar}), 128.5 (C_{Ar}), 128.4 (C_{Ar}), 128.0 (C_{Ar}), 127.1 (C_{Ar}), 61.1 ($CO_2CH_2CH_3$), 56.8 (Ph*C*HCO_2Et), 28.4 (PhCH_2CH_3), 15.4 (PhCH_2CH_3), 14.2 ($CO_2CH_2CH_3$). **HMRS (ESI)**: Mass calculated for $C_{18}H_{20}O_2Na$ $[M+Na]^+$, 291.1361. Found $[M+Na]^+$, 291.1357.

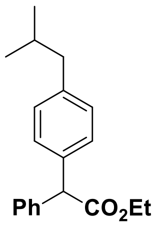

Ethyl 2-(4-isobuthylphenyl)-2-phenylacetate **7** (58 mg, 78% yield). **^{1}H NMR** (400 MHz, $CDCl_3$): δ 7.35-7.27 (m, 5H, CH_{Ar}), 7.28-7.18 (m, 2H, CH_{Ar}), 7.08 (d, 2H, CH_{Ar}), 4.97 (s, 1H, Ph*CH*CO_2Et), 4.19 (q, 2H, *J* = 7.1 Hz, $CO_2CH_2CH_3$), 2.43 (d, 2H, *J* = 7.6 Hz, $CH_2CH(CH_3)_2$), 1.83 (m, 1H, *J* = 7.6 Hz, $CH_2CH(CH_3)_2$), 1.25 (t, 3H, *J* = 7.1 Hz, $CO_2CH_2CH_3$), 0.88 (d, 6H, *J* = 7.6 Hz, $CH_2CH(CH_3)_2$). **^{13}C{^{1}H} NMR** (100 MHz, $CDCl_3$): δ 172.6 (*C*=O), 140.6 (C_{Ar}), 138.9 (C_{Ar}), 135.9 (C_{Ar}), 129.7 (C_{Ar}), 128.5 (C_{Ar}), 128.4 (C_{Ar}), 128.2 (C_{Ar}), 127.1 (C_{Ar}), 61.1 ($CO_2CH_2CH_3$), 56.7 (Ph*C*HCO_2Et), 45.0 ($CHCH_2(CH_3)_2$), 30.1 ($CHCH_2(CH_3)_2$), 22.3 ($CHCH_2(CH_3)_2$), 14.1 ($CO_2CH_2CH_3$). **HMRS (ESI)**: Mass calculated for $C_{20}H_{24}O_2Na$ $[M+Na]^+$, 319.1669. Found $[M+Na]^+$, 319.1667.

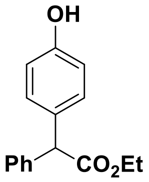

Ethyl 2-(4-hydroxyphenyl)-2-phenylacetate **8** (62 mg, 96% yield). **^{1}H NMR** (400 MHz, $CDCl_3$): δ 7.40-7.20 (m, 5H, CH_{Ar}), 7.14 (d, 2H, CH_{Ar}), 6.72 (d, 2H, CH_{Ar}), 5.81 (bs, 1H, O*H*Ph), 4.95 (s, 1H, Ph*CH*CO_2Et), 4.20 (q, 2H, *J* = 7.1 Hz, $CO_2CH_2CH_3$), 1.23 (t, 3H, *J* = 7.1 Hz, $CO_2CH_2CH_3$). **^{13}C{^{1}H} NMR** (100 MHz, $CDCl_3$): δ 173.3 (*C*=O), 155.0 (C_{Ar}), 155.1 (C_{Ar}), 138.9 (C_{Ar}), 129.8 (C_{Ar}), 128.6 (C_{Ar}), 128.5 (C_{Ar}), 128.4 (C_{Ar}), 127.1 (C_{Ar}), 115.5 (C_{Ar}), 61.3 ($CO_2CH_2CH_3$), 56.4 (Ph*C*HCO_2Et), 14.1 ($CO_2CH_2CH_3$).

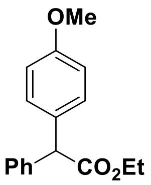

Ethyl 2-(4-methoxyphenyl)-2-phenylacetate **9** (67 mg, 99% yield). **^{1}H NMR** (400 MHz, $CDCl_3$): δ 7.32 (m, 5H, CH_{Ar}), 7.25 (d, 2H, CH_{Ar}), 6.86 (d, 2H, CH_{Ar}), 4.97 (s, 1H, PhC*H*CO_2Et), 4.21 (q, 2H, J = 7.1 Hz, $CO_2CH_2CH_3$), 3.78 (s, 3H, OCH_3Ph), 1.26 (t, 3H, J = 7.1 Hz, $CO_2CH_2CH_3$). **^{13}C{^{1}H} NMR** (100 MHz, $CDCl_3$): δ 172.7 (*C*=O), 158.7 (C_{Ar}), 139.1 (C_{Ar}), 130.9 (C_{Ar}), 129.6 (C_{Ar}), 128.5 (C_{Ar}), 128.4 (C_{Ar}), 127.1 (C_{Ar}), 113.9 (C_{Ar}), 61.1 ($CO_2CH_2CH_3$), 56.7 (Ph*C*HCO_2Et), 55.2 (OCH_3Ph), 14.1 ($CO_2CH_2CH_3$). **HMRS (ESI)**: Mass calculated for $C_{17}H_{18}O_3Na$ [*M*+Na]$^+$, 293.1154. Found [*M*+Na]$^+$, 293.1151.

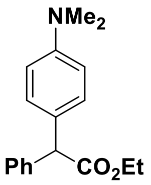

Ethyl 2-(4-(dimethylamino)phenyl)-2-phenylacetate **10** (70 mg, 99% yield). **^{1}H NMR** (400 MHz, $CDCl_3$): δ 7.33 (m, 4H, CH_{Ar}), 7.28 (m, 1H, CH_{Ar}), 7.23 (d, 2H, CH_{Ar}), 6.73 (d, 2H, CH_{Ar}), 4.97 (s, 1H, PhC*H*CO_2Et), 4.23 (q, 2H, J = 7.1 Hz, $CO_2CH_2CH_3$), 2.97 (s, 3H, CH_3Ph), 1.28 (t, 3H, J = 7.1 Hz, $CO_2CH_2CH_3$). **^{13}C{^{1}H} NMR** (100 MHz, $CDCl_3$): δ 173.1 (*C*=O), 149.3 (C_{Ar}), 139.5 (C_{Ar}), 129.3 (C_{Ar}), 128.4 (C_{Ar}), 127.1 (C_{Ar}), 126.9 (C_{Ar}), 112.8 (C_{Ar}), 61.0 ($CO_2CH_2CH_3$), 56.2 (Ph*C*HCO_2Et), 48.8 (CH_3NPh), 14.1 ($CO_2CH_2CH_3$).

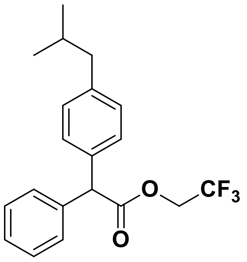

2,2,2-Trifluoroethyl 2-(4-isobutylphenyl)-2-phenylacetate **11** (80 mg, 91% yield) **^{1}H NMR** (400 MHz, $CDCl_3$): δ 7.24 (m, 5H, CH_{Ar}), 7.13 (d, 2H, J = 7.6 Hz, CH_{Ar}), 7.03 (m, 2H, J = 7.6 Hz, CH_{Ar}), 5.01 (s, 1H, C*H*$CO_2CH_2CF_3$), 4.51 (dq, 1H, J = 7.6 Hz, CO_2C*H*HCF_3), 4.43 (dq, 1H, J = 7.6 Hz, CO_2CH*H*CF_3), 2.43 (d, 2H, J = 7.1 Hz, CH$CH_2(CH_3)_2$), 1.83 (hept, 1H, J = 7.1 Hz, C*H*$CH_2(CH_3)_2$), 0.88 (d, 6H, J = 7.6 Hz, $CHCH_2(CH_3)_2$). **^{13}C{^{1}H} NMR** (100 MHz, $CDCl_3$): δ 170.8 (*C*=O), 1414 (C_{Ar}), 136.9 (C_{Ar}), 134.3 (C_{Ar}), 131.8 (C_{Ar}), 130.2 (C_{Ar}), 129.6 (C_{Ar}), 128.0 (C_{Ar}), 124.1 (C_{Ar}), 121.6 (C_{Ar}), 121.4 (C_{Ar}), 60.7 (q, 1C, $J_{C\text{-}F}$ = 34.9 Hz, $CO_2CH_2CF_3$), 55.5 (*C*H$CO_2CH_2CF_3$), 44.9 (*C*H$CH_2(CH_3)_2$), 30.1 (CH$CH_2(CH_3)_2$), 22.3 ($CHCH_2(CH_3)_2$). **^{19}F NMR** (375 MHz, $CDCl_3$): δ -73.6 (t, 3F, $J_{F\text{-}H}$ = 8.5 Hz, CH_2CF_3). **HMRS (ESI)**: Mass calculated for $C_{20}H_{21}O_2F_3Na$ [*M*+Na]$^+$, 373.1391. Found [*M*+Na]$^+$, 373.1382.

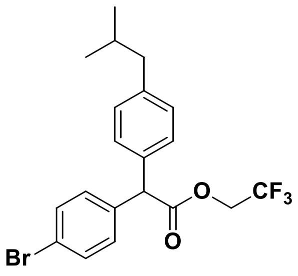

2,2,2-Trifluoroethyl-2-(4-bromophenyl)-2-(4-isobutylphenyl) acetate **12** (102 mg, 95% yield) **^{1}H NMR** (400 MHz, $CDCl_3$): δ δ 7.45 (d, 2H, CH_{Ar}), 7.44 (d, 2H, J = 8.0 Hz, CH_{Ar}), 7.18 (m, 5H, CH_{Ar}), 7.10 (d, 2H, CH_{Ar}), 5.01 (s, 1H, C*H*$CO_2CH_2CF_3$), 4.47 (q, 2H, J = 7.6 Hz, $CO_2CH_2CF_3$), 2.37 (d, 2H, J = 7.6 Hz, CH$CH_2(CH_3)_2$), 1.77 (hept, 1H, J = 7.6 Hz, C*H*$CH_2(CH_3)_2$), 0.81 (d, 6H, J = 7.6 Hz, $CHCH_2(CH_3)_2$). **^{13}C{^{1}H} NMR** (100 MHz, $CDCl_3$): δ 171.2 (*C*=O), 141.1 (C_{Ar}), 137.9 (C_{Ar}), 134.9 (C_{Ar}), 129.5 (C_{Ar}), 129.1 (C_{Ar}), 128.7 (C_{Ar}), 128.5 (C_{Ar}), 128.2 (C_{Ar}), 127.5 (C_{Ar}), 124.3 (C_{Ar}), 60.7 (q, 1C, $J_{C\text{-}F}$ = 36.6 Hz, $CO_2CH_2CF_3$), 56.2 ($CHCO_2CH_2CF_3$), 45.0 ($CHCH_2(CH_3)_2$), 30.1 ($CHCH_2(CH_3)_2$), 22.4 ($CHCH_2(CH_3)_2$). **^{19}F**

NMR (375 MHz, $CDCl_3$): δ -73.5 (t, 3F, $J_{F\text{-}H}$ = 8.6 Hz, CH_2CF_3). **HMRS (ESI)**: Mass calculated for $C_{20}H_{20}O_2F_3BrNa$ [M+Na]$^+$, 451.0496. Found [M+Na]$^+$, 451.0479.

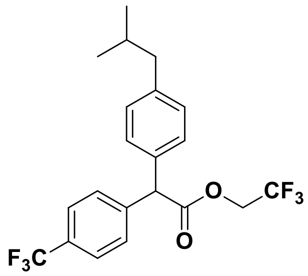

2,2,2–Trifluoroethyl-2-(4-isobutylphenyl)-2-(4- (trifluoromethyl)phenyl)acetate **13** (102 mg, 97% yield) **^{1}H NMR** (400 MHz, $CDCl_3$): δ 7.61 (d, 2H, J = 8.07 Hz, CH_{Ar}), 7.44 (d, 2H, J = 8.07 Hz, CH_{Ar}), 7.20 (d, 2H, J = 8.0 Hz, CH_{Ar}), 7.13 (d, 2H, J = 8.0 Hz, CH_{Ar}), 5.15 (s, 1H, $CHCO_2CH_2CF_3$), 4.56 (dq, 1H, J = 7.6 Hz, CO_2CHHCF_3), 4.55 (dq, 1H, J = 7.6 Hz, CO_2CHHCF_3), 2.46 (d, 2H, J = 7.8 Hz, $CHCH_2(CH_3)_2$), 1.85 (hept, 1H, J = 7.8 Hz, $CHCH_2(CH_3)_2$), 0.90 (t, J = 7.6 Hz, 3H, $CO_2CH_2CH_3$), 0.88 (d, 6H, J = 7.8 Hz, $CHCH_2(CH_3)_2$). **^{13}C{^{1}H} NMR** (100 MHz, $CDCl_3$): δ 170.5 (C=O), 141.7 (C_{Ar}), 141.5 (C_{Ar}), 133.9 (C_{Ar}), 129.6 (C_{Ar}), 128.9 (C_{Ar}), 128.0 (C_{Ar}), 125.7 (C_{Ar}), 125.6 (C_{Ar}), 125.5 (C_{Ar}), 125.4 (C_{Ar}), 121.3 (C_{Ar}), 61.1 (q, 1C, $J_{C\text{-}F}$ = 35.8 Hz, $CO_2CH_2CF_3$), 55.9 ($CHCO_2CH_2CF_3$), 45.0 ($CHCH_2(CH_3)_2$), 30.1 ($CHCH_2(CH_3)_2$), 22.3 ($CHCH_2(CH_3)_2$). **^{19}F NMR** (375 MHz, $CDCl_3$): δ -62.6 (s, 3F, CF_3-Ph), -73.6 (t, 3F, $J_{F\text{-}H}$ = 8.6 Hz, CH_2CF_3). **HMRS (ESI)**: Mass calculated for $C_{21}H_{20}O_2F_6Na$ [M+Na]$^+$, 441.1265. Found [M+Na]$^+$, 441.1255.

4. NMR Spectra.

Ethyl 2-phenyl-2-(p-tolyl)acetate **2**

^{1}H NMR (400 MHz, $CDCl_3$)

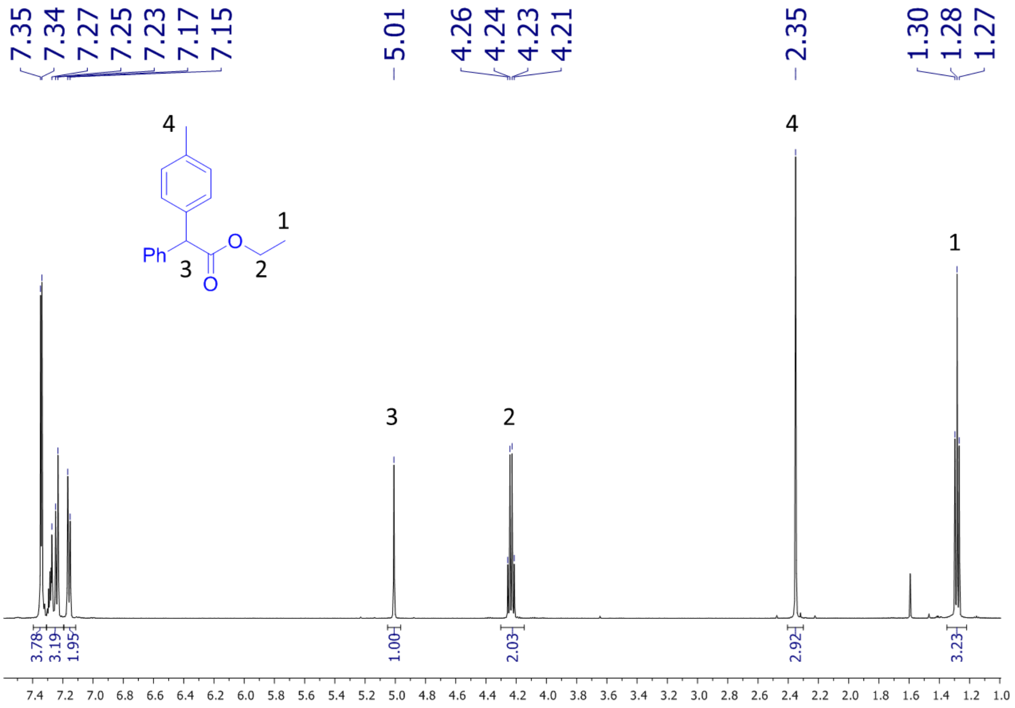

$^{13}C\{^1H\}$ NMR (100 MHz, $CDCl_3$)

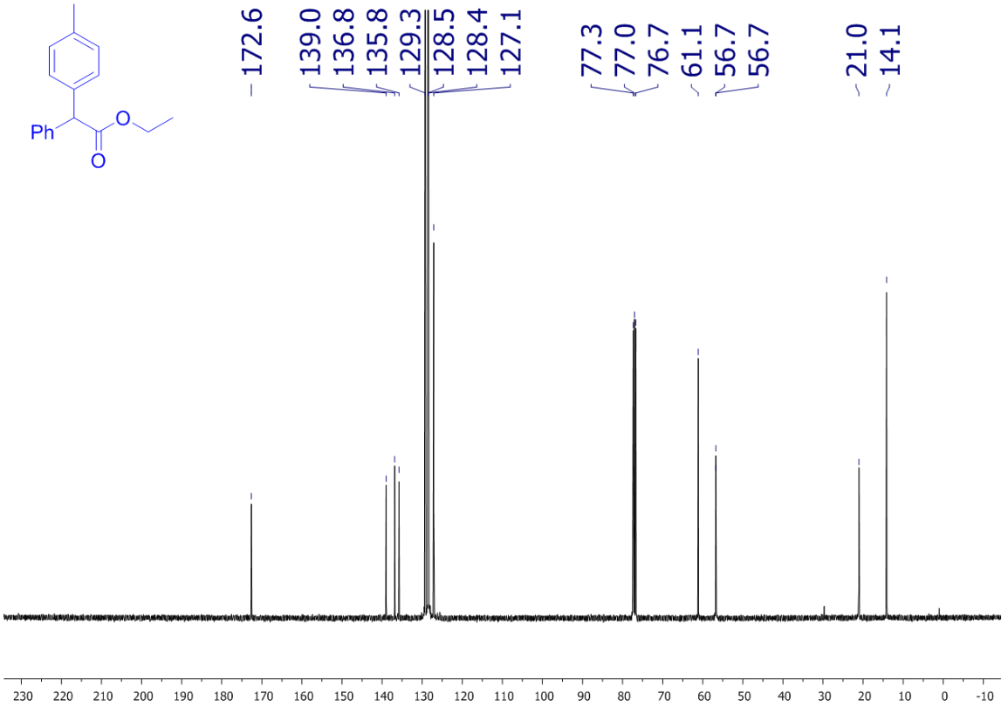

Ethyl 2-(4-ethylphenyl)-2-phenylacetate **6**

^{1}H NMR (400 MHz, $CDCl_3$)

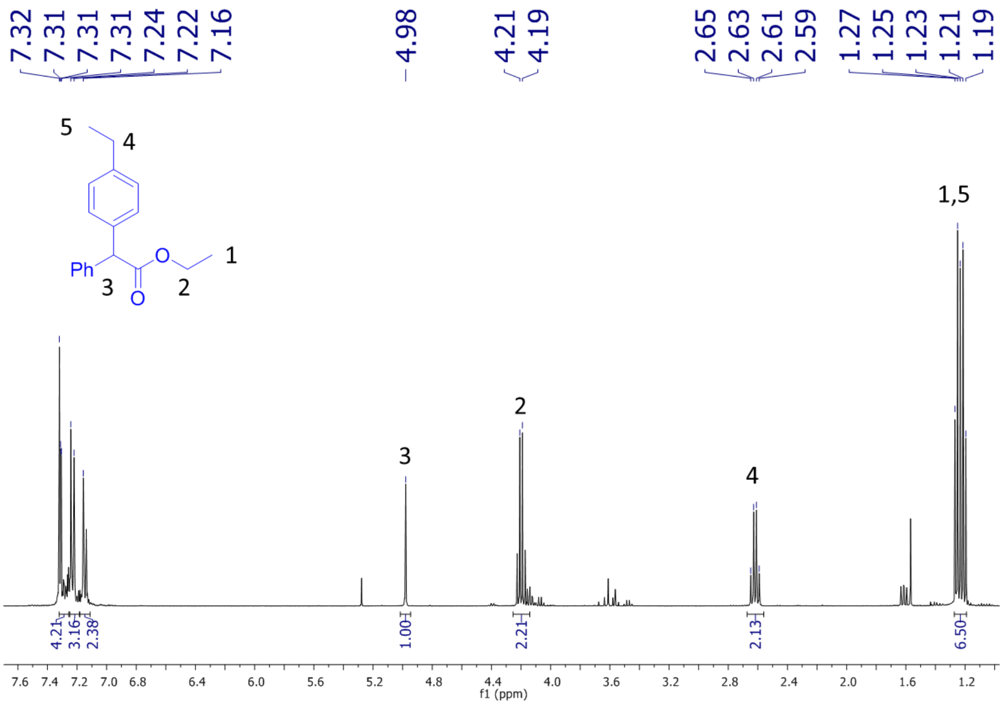

$^{13}C\{^{1}H\}$ NMR (100 MHz, $CDCl_3$)

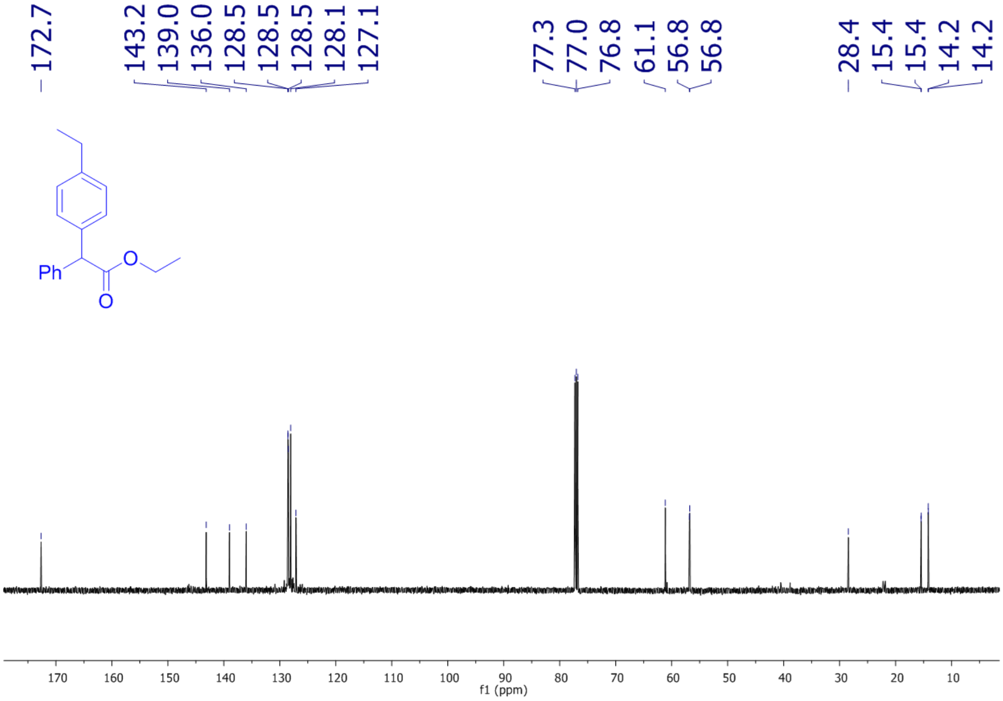

Ethyl 2-(4-isobuthylphenyl)-2-phenylacetate **7**

^{1}H NMR (400 MHz, $CDCl_3$)

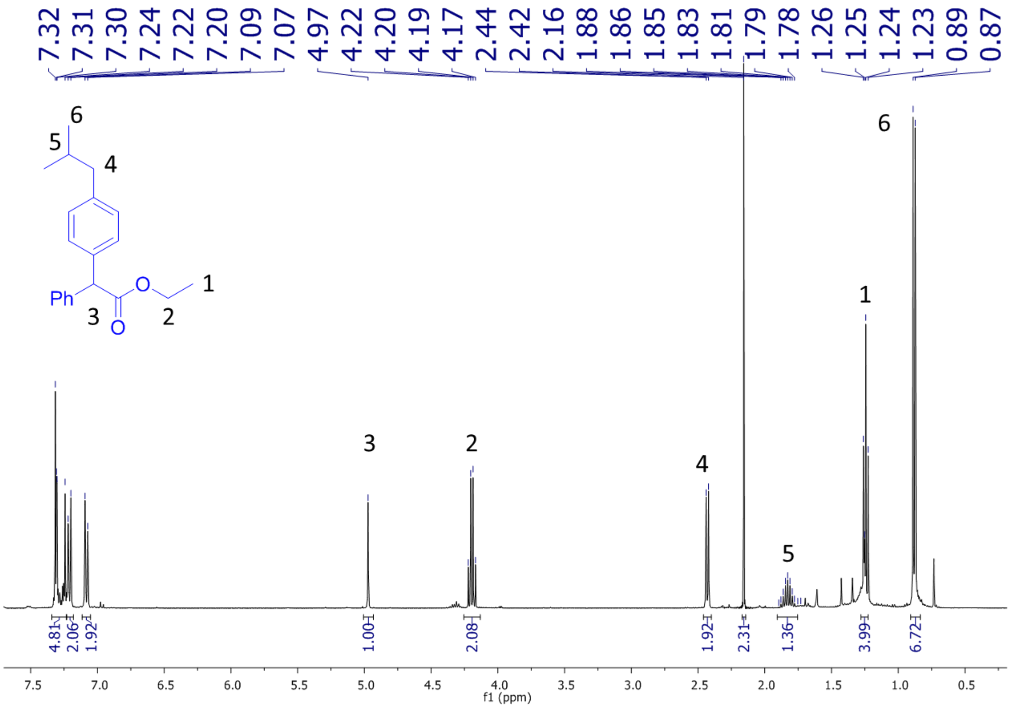

$^{13}C\{^{1}H\}$ NMR (100 MHz, $CDCl_3$)

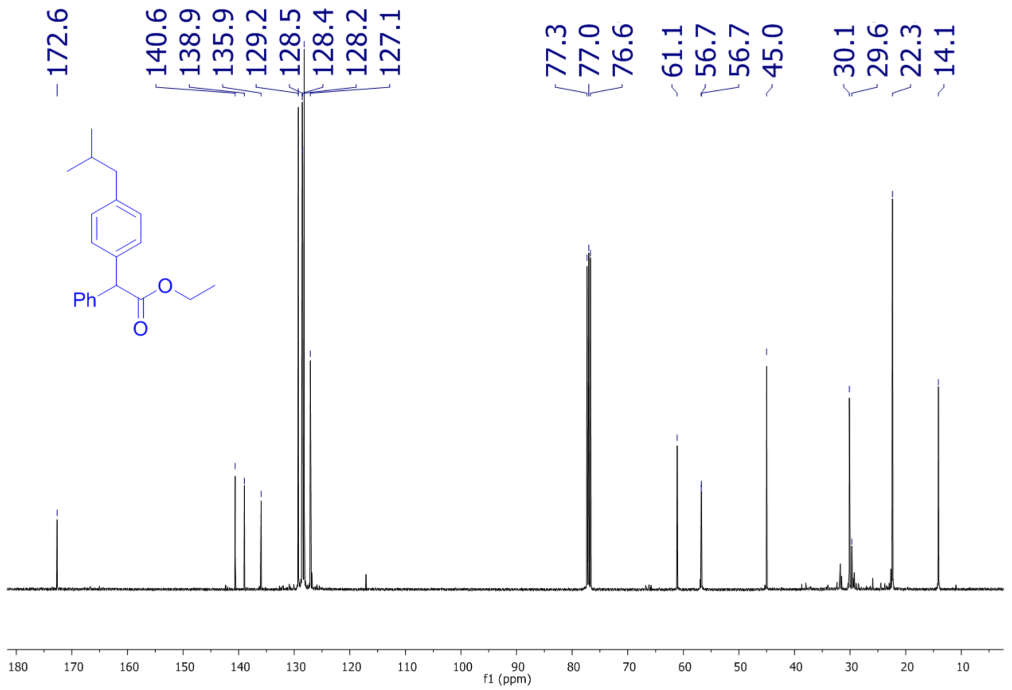

Ethyl 2-(4-hydroxyphenyl)-2-phenylacetate **8**

^{1}H NMR (400 MHz, $CDCl_3$)

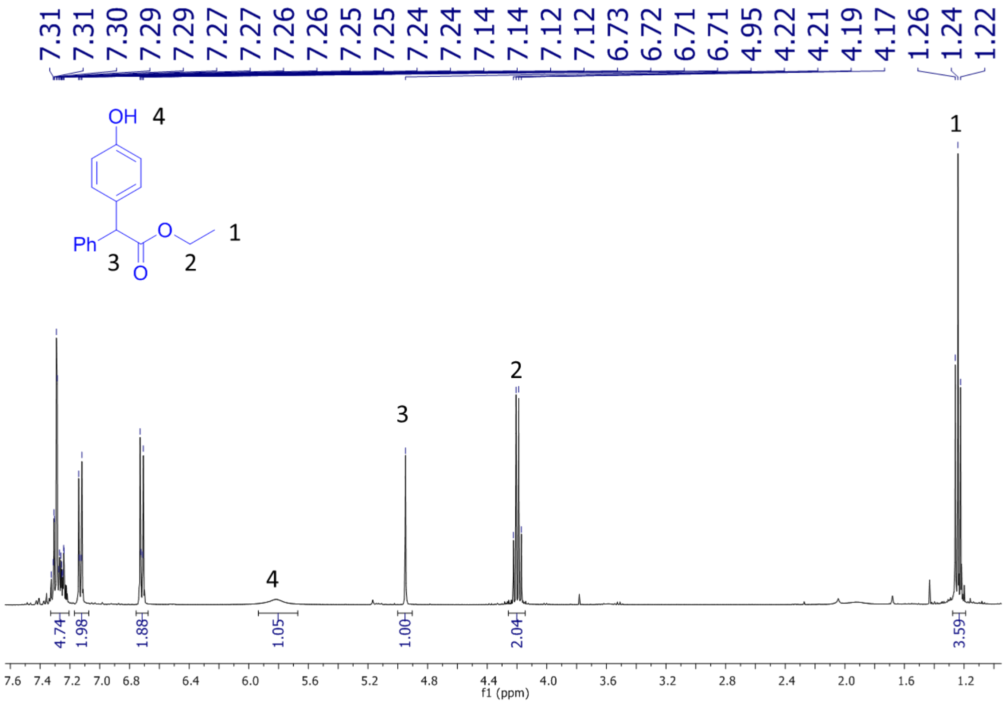

$^{13}C\{^1H\}$ NMR (100 MHz, $CDCl_3$)

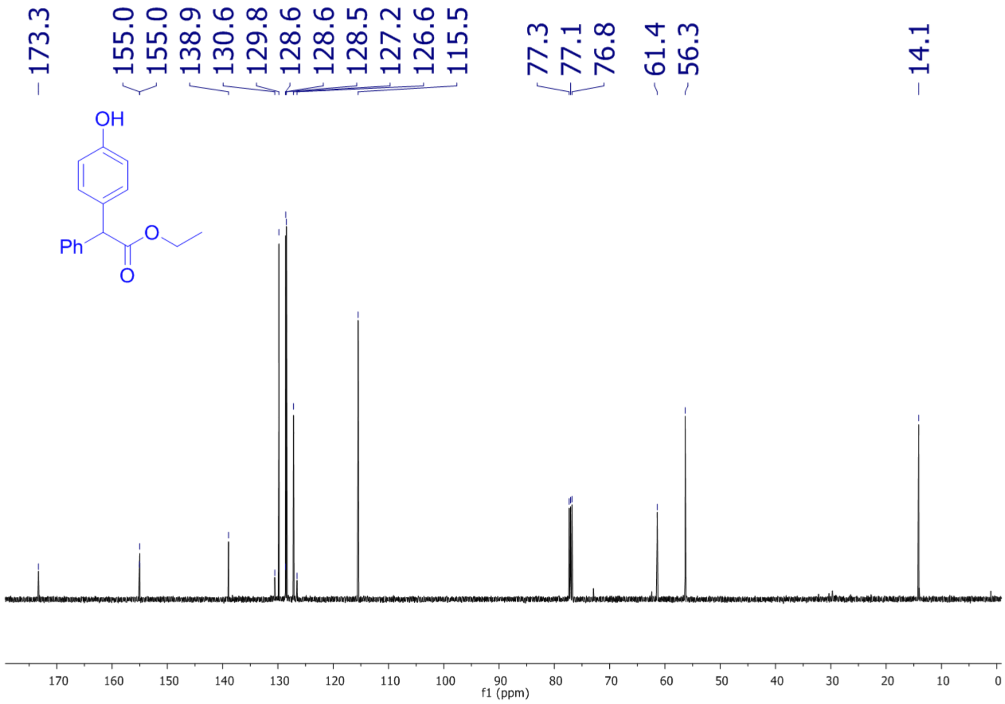

Ethyl 2-(4-(dimethylamino)phenyl)-2-phenylacetate **9**

^{1}H NMR (400 MHz, $CDCl_3$)

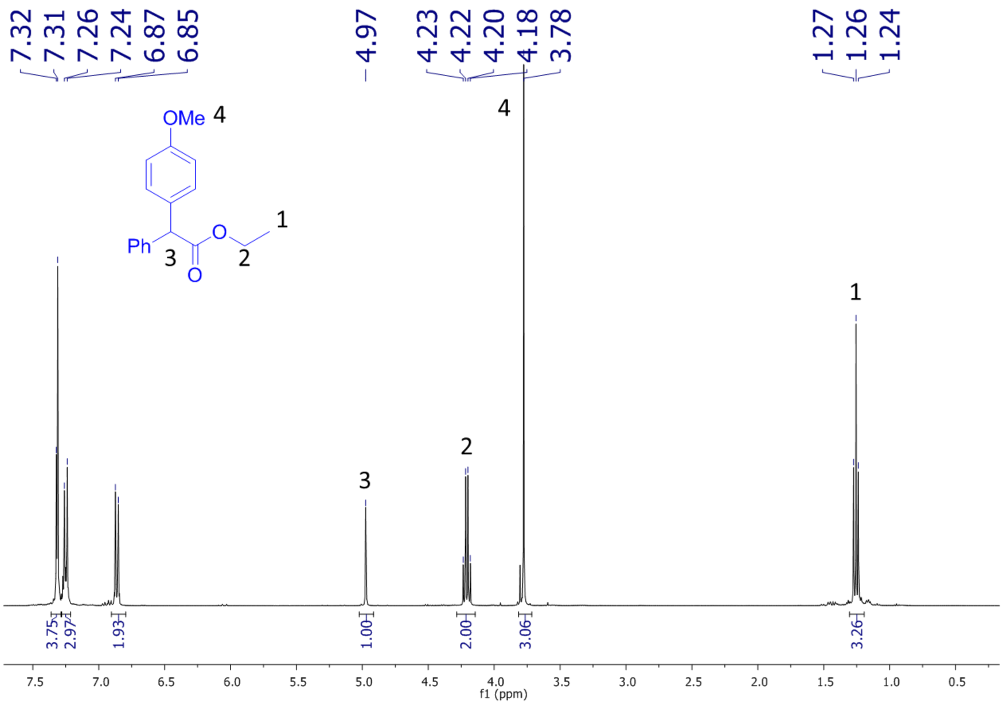

$^{13}C\{^{1}H\}$ NMR (100 MHz, $CDCl_3$)

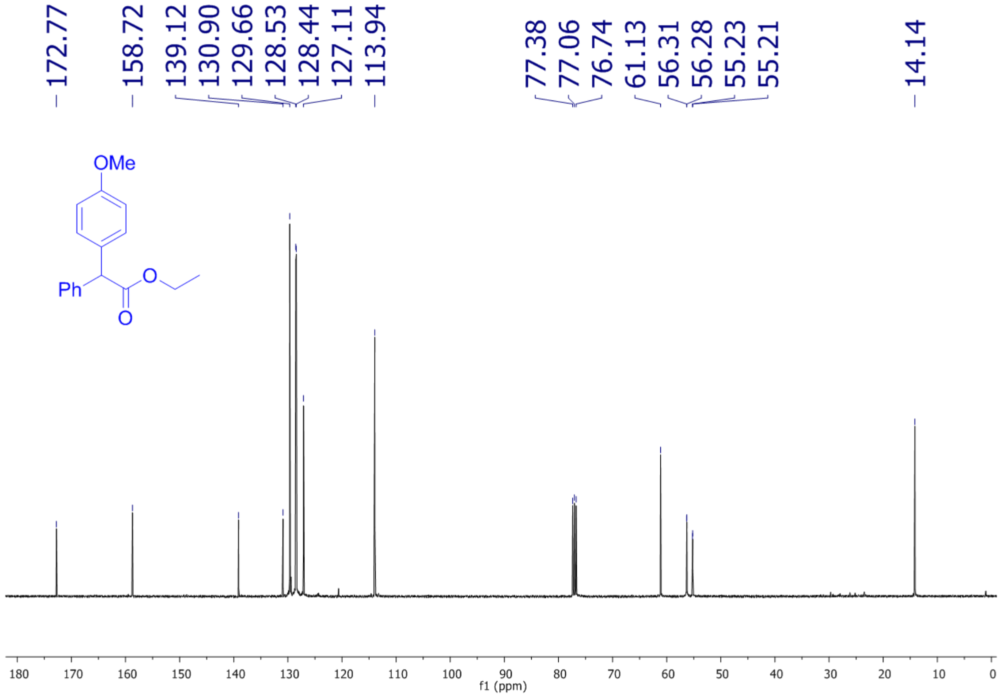

Ethyl 2-(4-(dimethylamino)phenyl)-2-phenylacetate **10**

^{1}H NMR (400 MHz, $CDCl_3$)

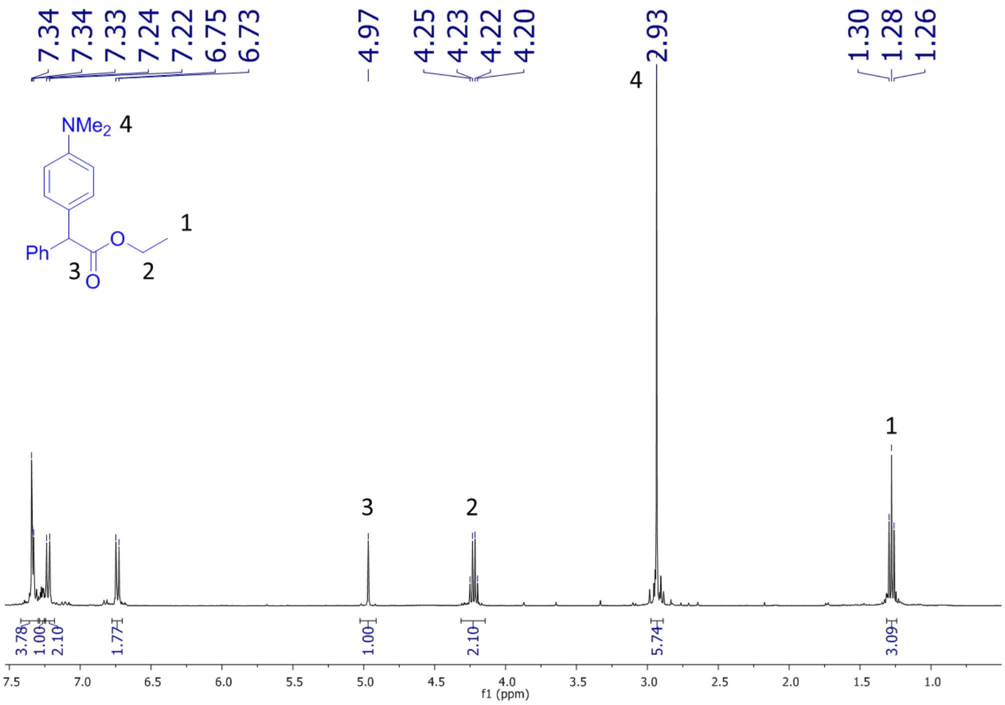

$^{13}C\{^{1}H\}$ NMR (100 MHz, $CDCl_3$)

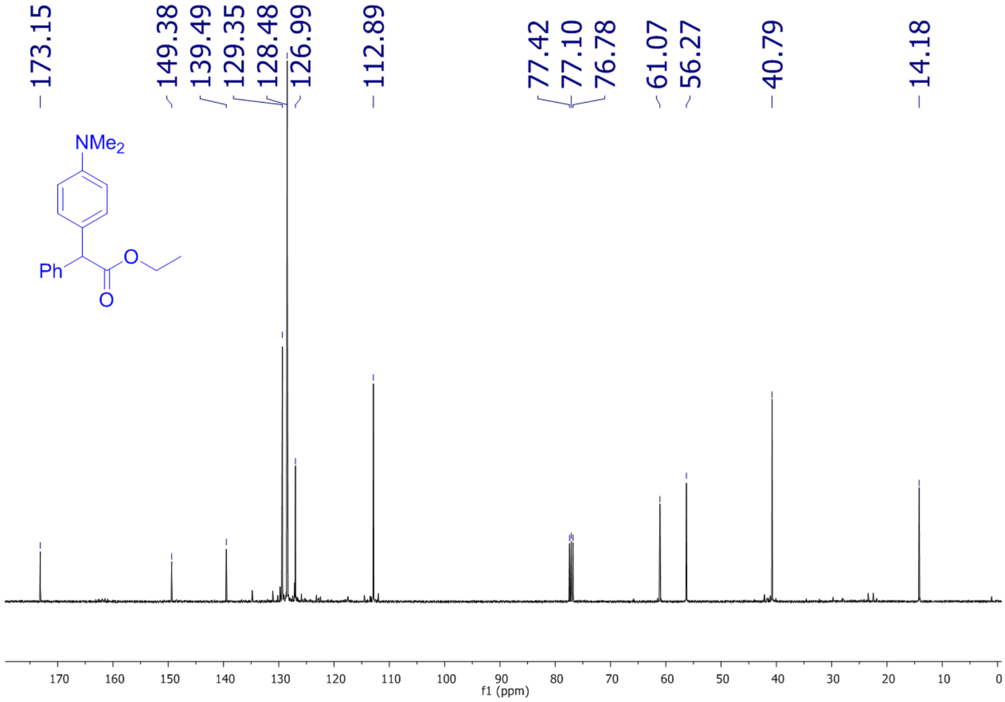

2,2,2-Trifluoroethyl 2-(4-isobutylphenyl)-2-phenylacetate **11**

^{1}H NMR (400 MHz, $CDCl_3$)

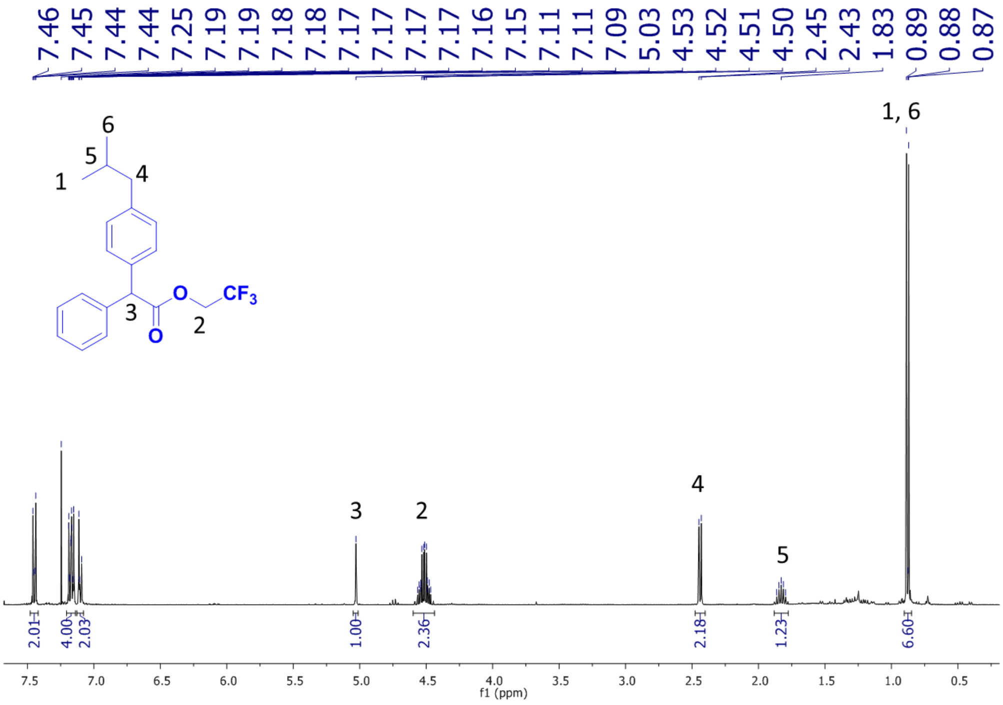

$^{13}C\{^1H\}$ NMR (100 MHz, $CDCl_3$)

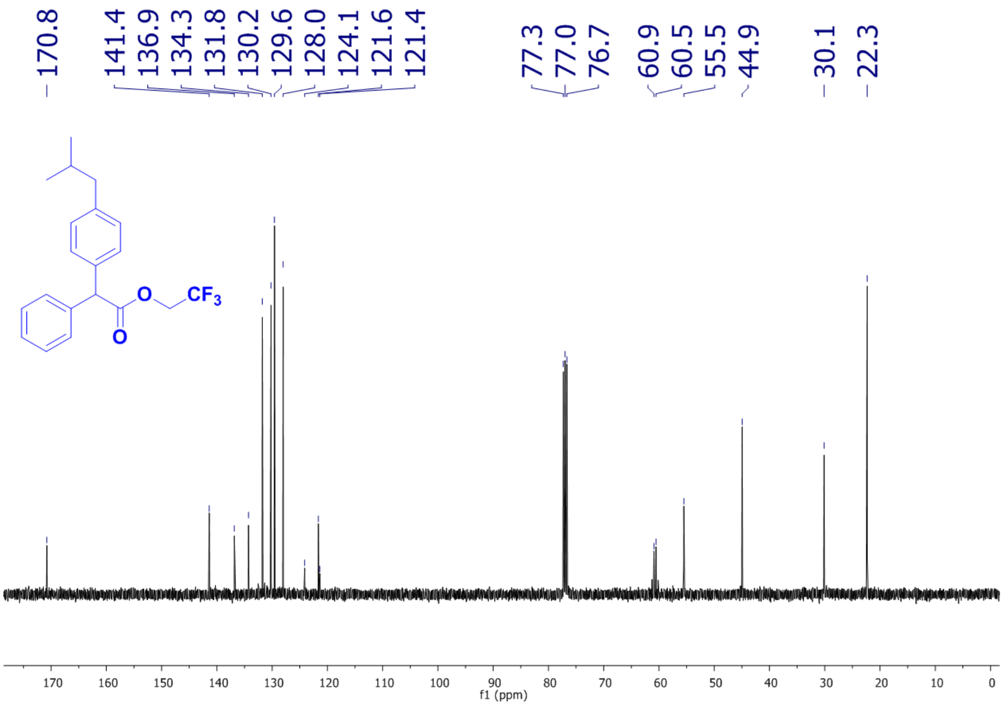

^{19}F NMR (375 MHz, $CDCl_3$)

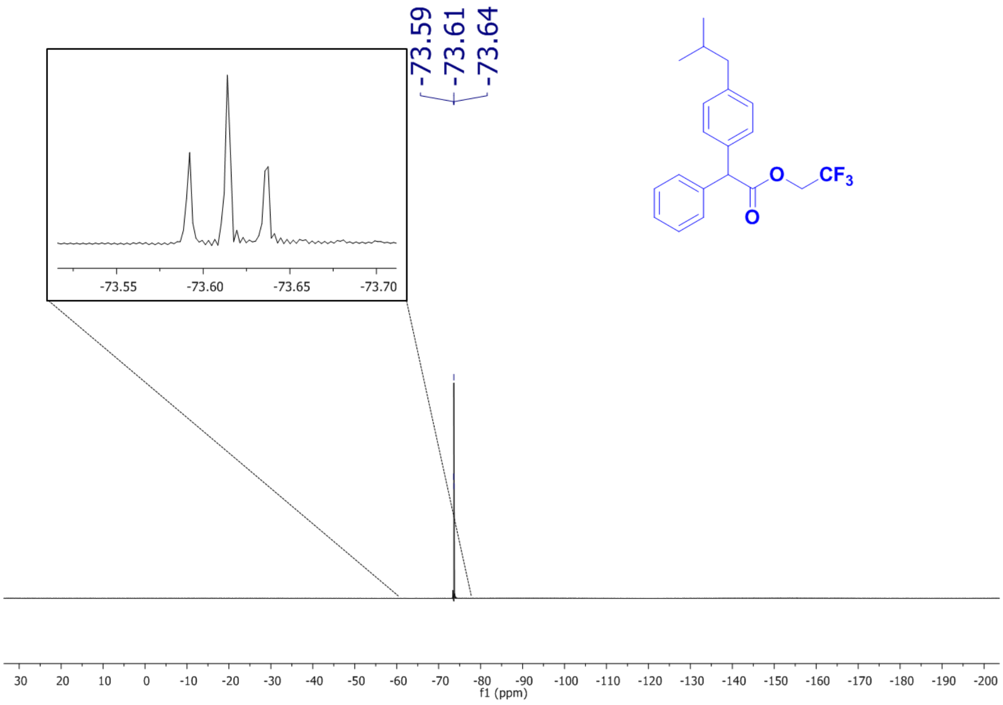

2,2,2-Trifluoroethyl-2-(4-bromophenyl)-2-(4-isobutylphenyl)acetate **12**

^{1}H NMR (400 MHz, $CDCl_3$)

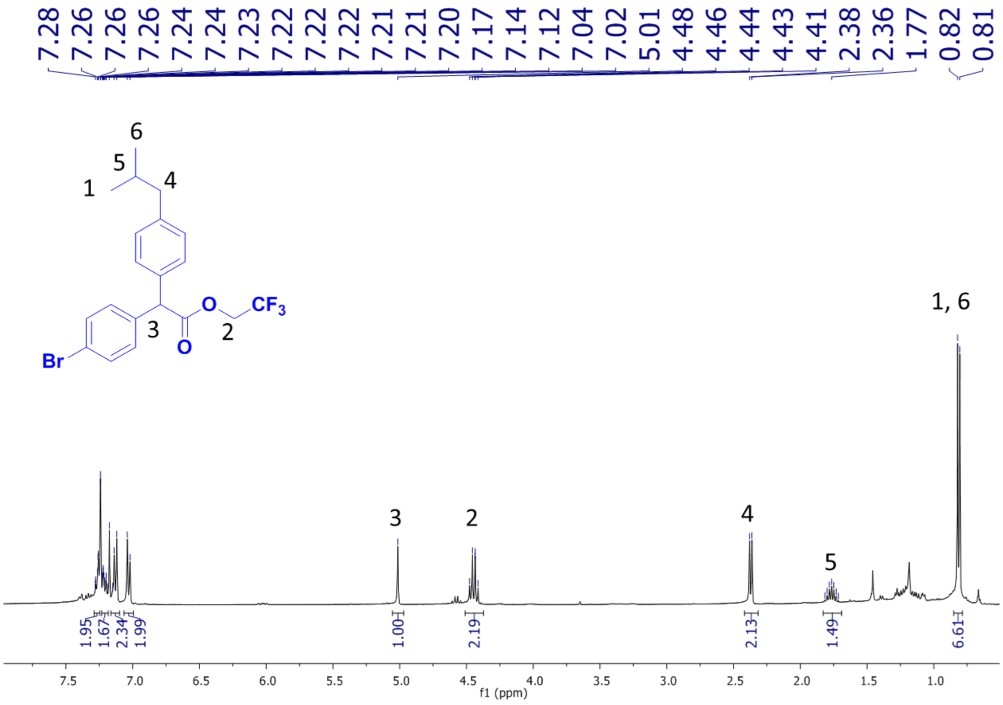

$^{13}C\{^{1}H\}$ NMR (100 MHz, $CDCl_3$)

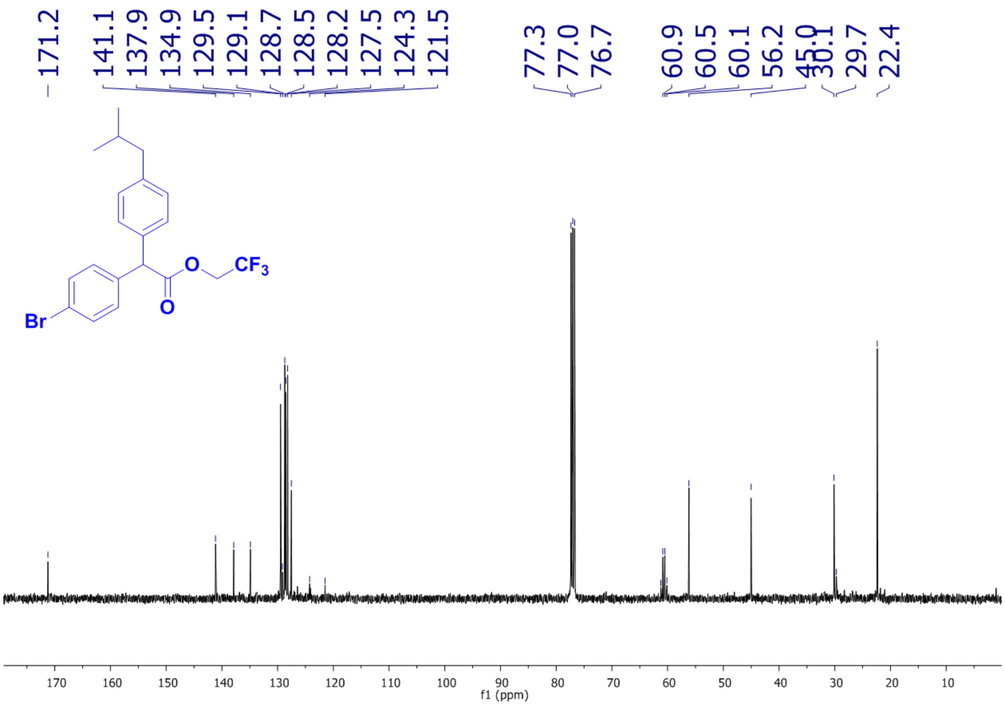

^{19}F NMR (375 MHz, $CDCl_3$)

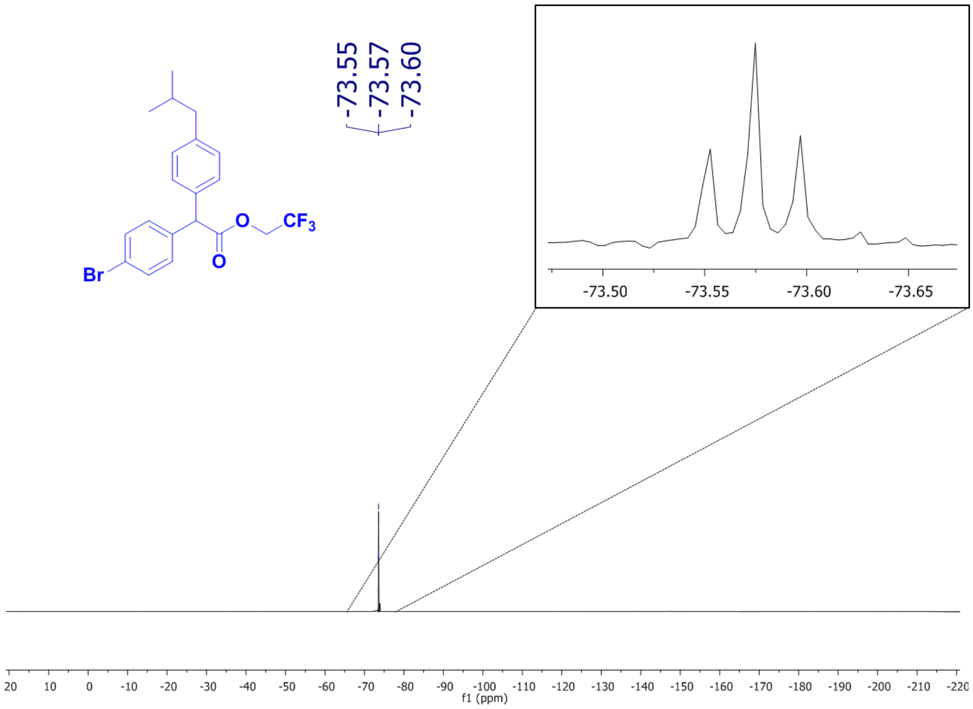

2,2,2–Trifluoroethyl-2-(4-isobutylphenyl)-2-(4- (trifluoromethyl)phenyl)acetate **13**

^{1}H NMR (400 MHz, $CDCl_3$)

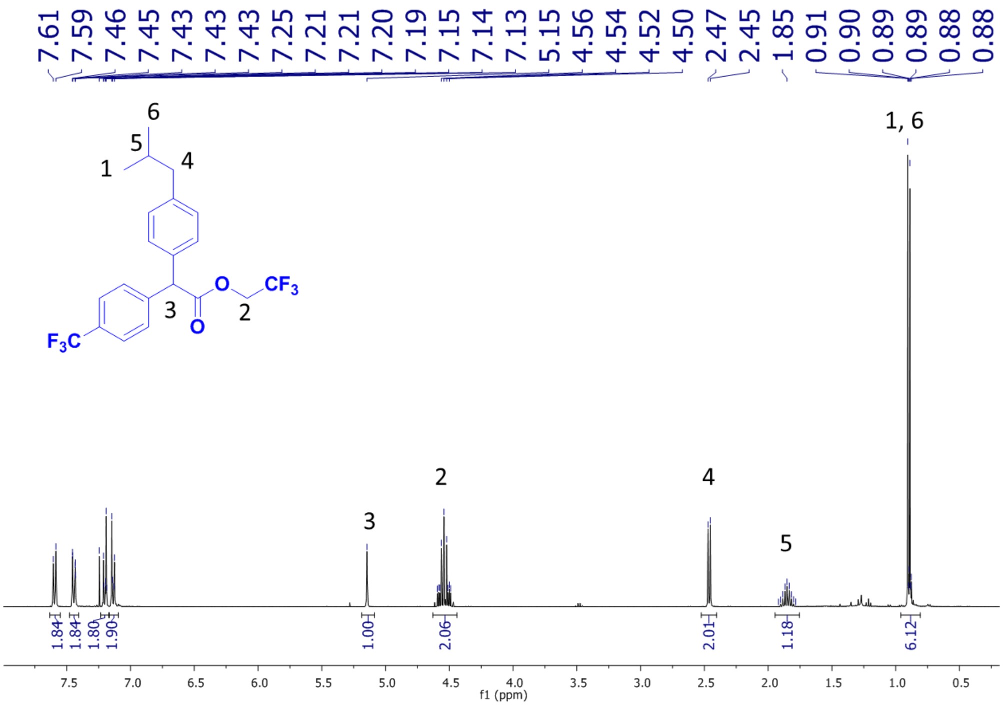

^{13}C{^{1}H} NMR (100 MHz, $CDCl_3$)

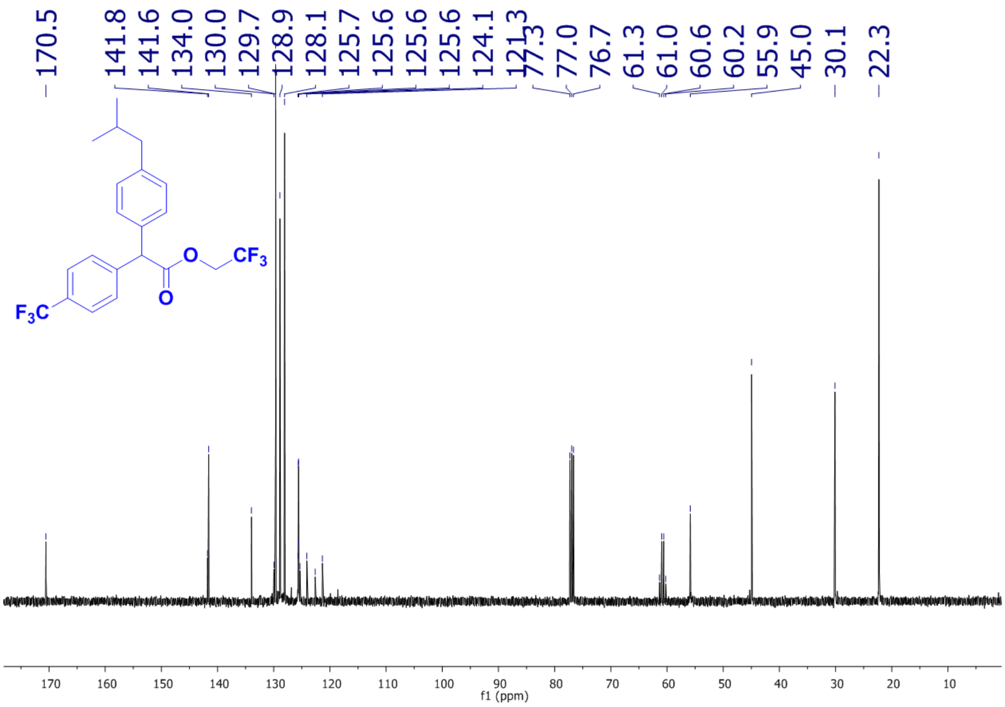

19F NMR (375 MHz, CDCl$_3$)

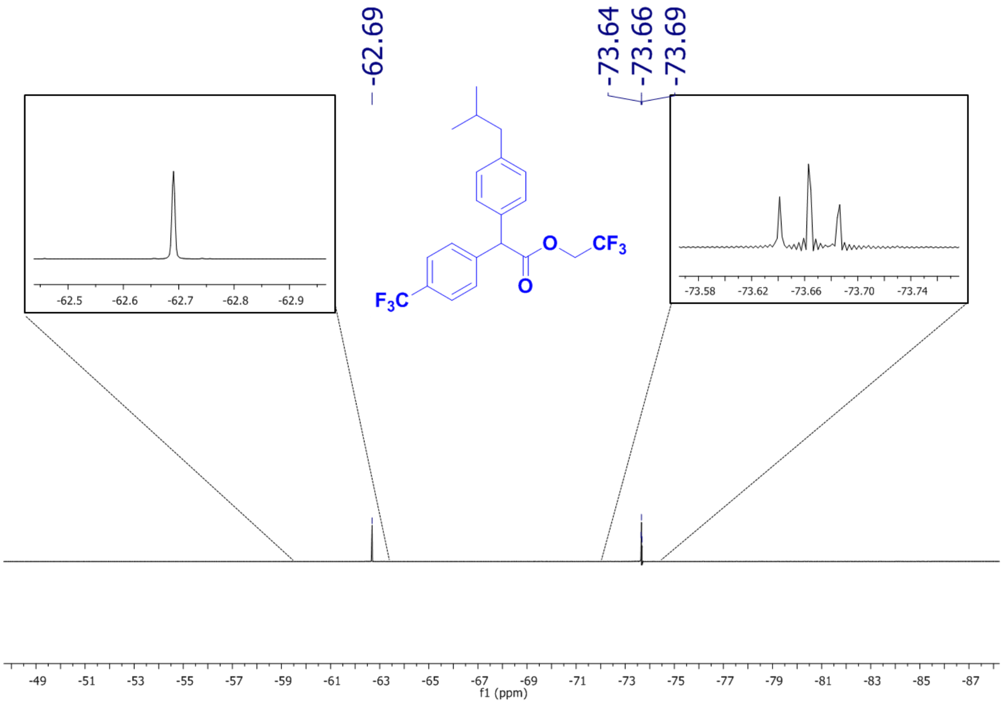

References

(1) Zhang, B.; Hollerbach, M. R.; Blakey, S. B.; Davies, H. M. L. C–H Functionalization Approach for the Synthesis of Chiral C2-Symmetric 1,5-Cyclooctadiene Ligands. *Org. Lett.* **2019**, *21*, 9864.

(2) Pizarro, J. D.; Molina, F.; Fructos, M. R.; Pérez, P. J. Alkoxydiaminophosphine Ligands as Surrogates of NHCs in Copper Catalysis. *Chem. Eur. J.* **2020**, *26*, 10330.

(3) Pizarro, J. D.; Molina, F.; Fructos, M. R.; Pérez, P. J. Gold Complexes with ADAP Ligands: Effect of Bulkiness in Catalytic Carbene Transfer Reactions (ADAP = Alkoxydiaminophosphine). *Organometallics* **2020**, *39*, 2553.

(4) Fructos, M. R.; Belderrain, T. R.; de Frémont, P.; Scott, N. M.; Nolan, S. P.; Díaz-Requejo, M. M.; Pérez, P. J. A Gold Catalyst for Carbene-Transfer Reactions from Ethyl Diazoacetate. *Angew. Chem. Int. Ed.* **2005**, *44*, 5284.

(5) Chen, T. Q.; MacMillan, D. W. C. A Metallaphotoredox Strategy for the Cross-Electrophile Coupling of α-Chloro Carbonyls with Aryl Halides. *Angew. Chem. Int. Ed.* **2019**, 58, 14584.

6. Computational details

DFT calculations were carried out with the hybrid PBE0 GGA functional, as implemented in the Gaussian16 software package. Structures were fully optimized without any geometry or symmetry constraints with the double-ζ quality def2SVP basis set. Vibrational frequencies were computed at the same level of theory to classify all stationary points as either saddle points (transition states, with a single imaginary frequency) or energy minima (reactants, intermediates and products, with only real frequencies). These calculations were also used to obtain the thermochemistry corrections (zero-point, thermal and entropy energies) at p = 1 atm and T = 298.15 K conditions. The energy of the optimized geometries was refined by single point calculations with the triple-ζ quality def2TZVP basis set. The energies reported in the manuscript were obtained by adding the thermochemistry corrections to the refined potential energies. Single-point calculations were used to investigate the electronic structure of the **I1a** and **I2a** complexes by means of natural bond orbital analysis with the NBO7 software.

Dispersion forces and solvent effects for dichloromethane were included in all calculations (geometry optimization, frequencies and single points) using different methods: GD3bj + SMD in **method I**, GD3 + CPCM in **method II**, and GD3 + SMD in **method III** (see Table S7). **Method II** was used for all of the mechanistic studies because calculations were faster and did not give convergence problems. However, we found that this method underestimates the difference in energy barriers ($\Delta\Delta G^{\ddagger}$) for the *para* and *ortho* addition. On the other hand, the most sophisticated **method I** gave results more consistent with experimental data but frequently failed during geometry optimization. Therefore, **method I** was used for computing the lowest energy pathways, which are those included in the manuscript, while **method II** was used for exploring different orientations of the carbene relative to toluene addition (see Figure S1).

Table S7. Free energy barriers (in kcal mol^{-1}) for the toluene addition to carbene in *para* and *ortho* using **methods I, II** and **III**.

**Method**	$1^{OEt-OEt}$	1^{OEt-O}	$\Delta G^{\ddagger}_{para}$	$\Delta G^{\ddagger}_{ortho}$	$\Delta\Delta G^{\ddagger}$
**I**	0.8	0	14.3	17.5	3.2
**II**	0	0.3	13.3	14.9	1.6
**III**	0	0.5	15.5	17.4	1.9

Addition of toluene to I1a-Ph considering different orientation of the carbene:

In this section, we have renamed intermediates to distinguish the different carbene orientations and to differentiate the structures obtained with the computational **method I** (used in the manuscript) from those calculated with **method II** (used in Figure S1 and S2).

Two different isomers of **I1a-Ph**, named $\mathbf{1}^{\mathbf{OEt\text{-}OEt}}$ and $\mathbf{1}^{\mathbf{OEt\text{-}O}}$, were found depending on the relative position of the acetate C-OEt and C=O with respect to the phosphine OEt group. These isomers yield four reactive pathways: two for the addition of toluene by the side of the phosphine OEt group, **OEt-OEt** and **OEt-O**, and two by the side of the N-substituents of the phosphine (**N-OEt** and **N-O**) (see Figure S1).

The small Free energy difference between $\mathbf{1}^{\mathbf{OEt\text{-}OEt}}$ and $\mathbf{1}^{\mathbf{OEt\text{-}O}}$ (ΔG=0.3 kcal mol^{-1}) and the low energy barrier connecting the two isomers ($\mathbf{TS1}^{\mathbf{rot}}$ =9.8 kcal mol^{-1}), indicate that both species will exist in solution, independently of which one is initially formed by N_2 elimination (See Figure S2). The energy barriers for the toluene addition in *para* position show that there is a small preference for the addition by the side of the OEt phosphine substituent, and the addition by the side of the acetate OEt. These preferences result in the following trends in the transition state energies: $\mathbf{TS}^{\mathbf{OEt\text{-}OEt}}$ < $\mathbf{TS}^{\mathbf{OEt\text{-}O}}$ < $\mathbf{TS}^{\mathbf{N\text{-}OEt}}$ < $\mathbf{TS}^{\mathbf{N\text{-}O}}$. However, toluene deprotonation is preferred if the C-H bond of toluene and the C=O group of acetate are on the same side. In that case, proton migration has an energy barrier of *ca* 1 kcal mol^{-1} (we estimated this barrier by using a scan calculation involving the dihedral angle θ) and is highly exergonic (-29.0 kcal mol^{-1} from $\mathbf{2}^{\mathbf{OEt\text{-}O}}$ to $\mathbf{3}^{\mathbf{OEt\text{-}O}}$). Otherwise, the deprotonation requires the rotation of the acetate group in intermediate $\mathbf{2}^{\mathbf{OEt\text{-}OEt}}$ ($\mathbf{TS2}^{\mathbf{rot}}$ = 14.7 kcal mol^{-1}) to yield $\mathbf{2}^{\mathbf{OEt\text{-}O}}$ and is nearly thermoneutral (0.8 kcal mol^{-1}).

An opposite trend is observed for the addition of toluene in the *ortho* position, in which $\mathbf{TS}^{\mathbf{OEt\text{-}O}}$ is slightly preferred over $\mathbf{TS}^{\mathbf{OEt\text{-}OEt}}$ by 0.6 kcal mol^{-1}, due to steric hindrance between the toluene-Me and acetate-OEt groups. Interestingly, the orientation of the methyl group towards the ADAP ligand (TSOEt-O/OEt-ortho-in, vide infra) is energetically favoured by 0.8 kcal mol^{-1} for $\mathbf{TS}^{\mathbf{OEt\text{-}OEt}}$ and by 2.1 kcal mol^{-1} for $\mathbf{TS}^{\mathbf{OEt\text{-}O}}$. More significantly, the rotation of the acetate group after the toluene *ortho*-addition has a higher energy barrier than in the *para*-addition (22.5 kcal mol^{-1} in $\mathbf{TS2}^{\mathbf{rot}}$***-ortho***, and 14.7 kcal mol^{-1} in $\mathbf{TS2}^{\mathbf{rot}}$***-para***). This energy difference implies that the toluene *ortho-para* selectivity is governed by the difference in energy of $\mathbf{TS}^{\mathbf{OEt\text{-}O}}$, which is 1.6 kcal mol^{-1} using **method II** (and 3.2 kcal mol^{-1} with **method I**). We think that this energy difference together with the endergonic addition of toluene are responsible for the *para* selectivity.

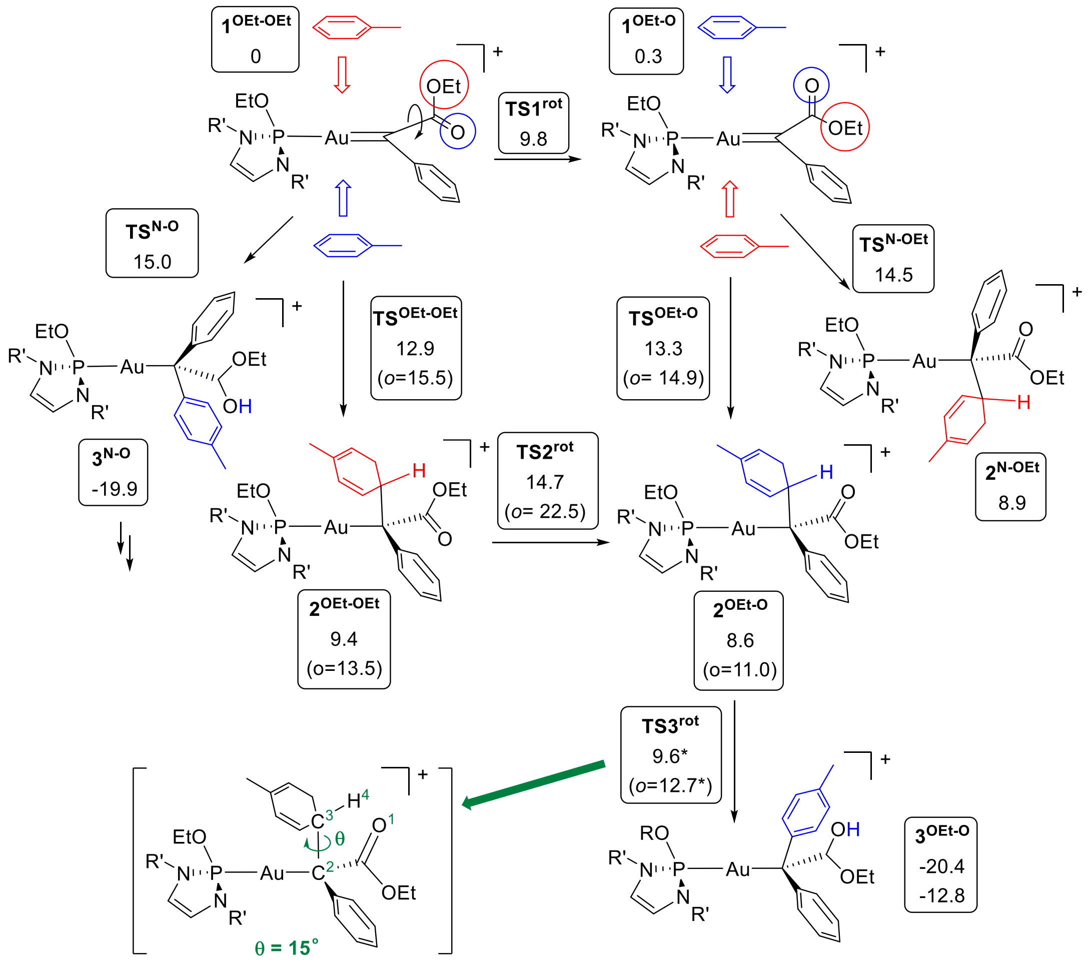

Figure S1: Free energy (in kcal mol^{-1}) for the steps defining regioselectivity in the functionalization of toluene with $1^{OEt\text{-}OEt}$ in *para* and *ortho* position with **method II**.

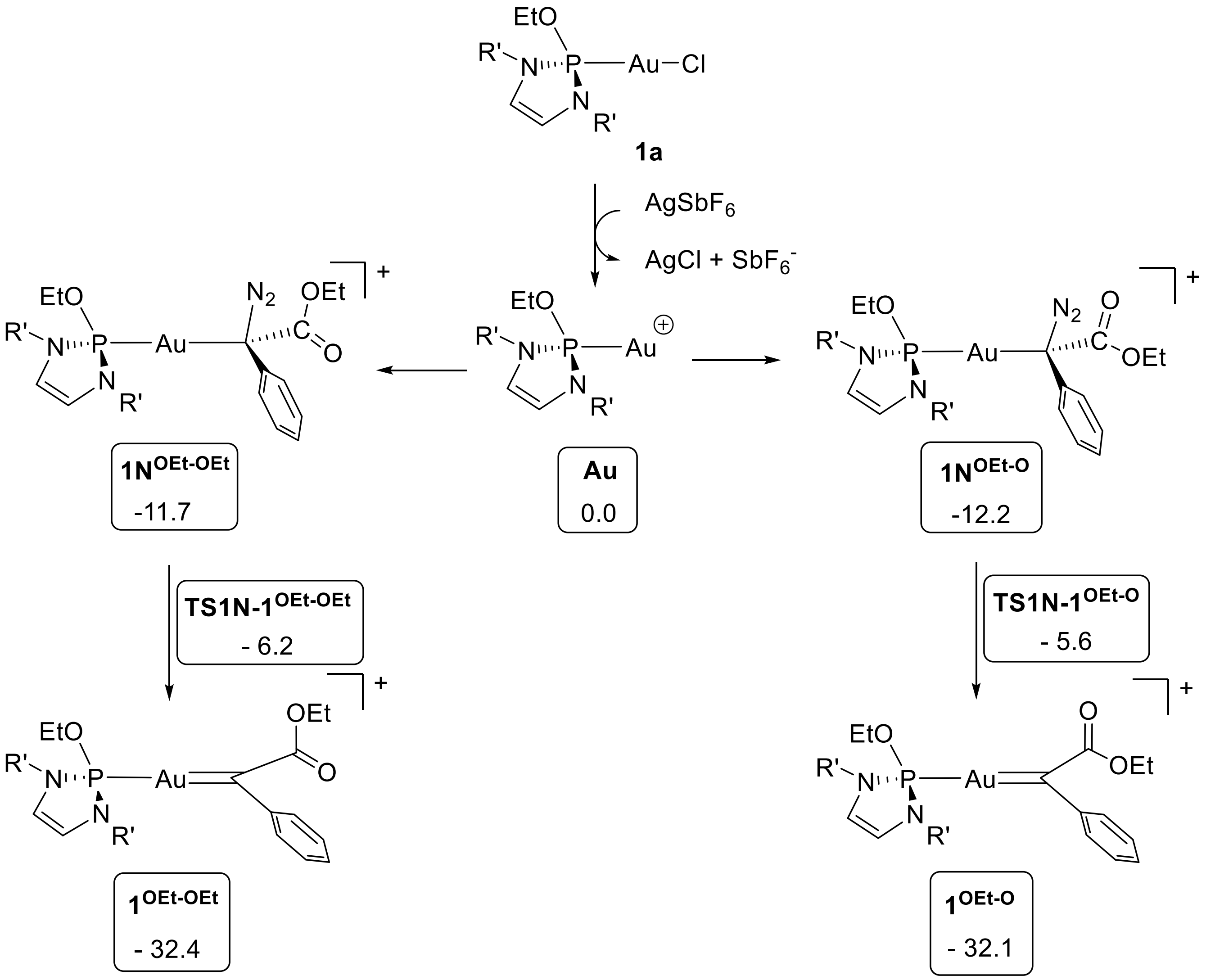

Figure S2: Free energies (in kcal mol^{-1}) for the formation of $\mathbf{1}^{\text{OEt-OEt}}$ and $\mathbf{1}^{\text{OEt-O}}$ from **1a** after removal of Cl with **method II**.

NBO analysis of I1a and I2a species

We performed NBO analysis on the **I1a** and **I2a** species with Ph and H substituents. The numerical parameters of these analyses are summarized in Table S8.

Overall, it was found that the net electron donation into the low vacancy orbital of the carbene (p_{C1}) is smaller in **I1a-H** compared to **I1a-Ph**, resulting in a more electrophilic carbon in **I1a-H**. For **I1a-Ph** the largest donation to the low vacancy orbital of the carbene originates from the phenyl group ($\pi_{C=C(Ph)} \rightarrow p_{C1}$, see also Figure S3). Furthermore, π-back-donation from the gold complex can be observed ($d_{Au} \rightarrow p_{C1}$, see also Figure S3). This π-back-donation is slightly stronger in **I1a-H** (see also Figure S4). Additionally, an unusual σ-donation of the carbonyl group into the low vacancy orbital of the carbene of **I1a-H** was found ($p_O \rightarrow p_{C1}$, see also Figure S4). This interaction affects the C1-C2=O angle in **I1a-H** which was found to be 102°. When modelling **I1a-H** with a restricted angle (α = 120°) the electron donation is less pronounced (5.8 kcal mol^{-1} compared to 28.1 kcal mol^{-1} without restriction, see also Figure S5). While the potential energy of the intermediate does not change significantly (ΔΔE = 2.6 kcal/mol), an energy decrease of the LUMO is observed, consistent with a higher reactivity of the **I1a-H** species towards the nucleophilic addition of toluene. Both **I1a** molecules show a strong donation from the carbonic carbon to the antibonding σ* orbital of the Au-P bond. This donation is more pronounced for **I1a-H** with 219.0 kcal mol^{-1} compared to 193.1 kcal mol^{-1} for **I1a-Ph**. The same donation is found in the **I2a** intermediates, increased for **I2a-H** compared to **I1a-H** and decreased for **I2a-Ph** compared to **I1a-Ph**.

Table S8: Structural and electronic parameters of the **I1a** and **I2a** species.

Compound	Name	Distances and angles	Natural Charges[a]	Donor-Acceptor Interactions[b]	Interactions Stabilization Energies[b,c]
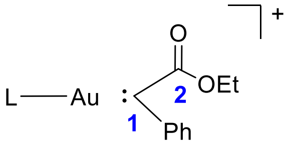 LUMO = -4.14 eV	I1a-Ph	2.03 Å (Au-C1)	-0.07 (C1)	$sp^{1.8}_{C1} \rightarrow \pi^*_{C2=O}$	22.9
				$sp^{1.8}_{C1} \rightarrow \pi^*_{C=C(Ph)}$	11.1
				$\pi_{C=C(Ph)} \rightarrow p_{C1}$	101.7
				$sp^{1.8}_{C1} \rightarrow \sigma^*_{Au-P}$	193.1
				$d_{Au} \rightarrow p_{C1}$	24.0
				$p_{O} \rightarrow p_{C1}$	4.2
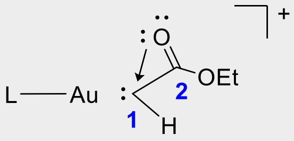 LUMO = -4.01 eV	I1a-H	1.99 Å (Au-C1)	-0.19 (C1)	$p_{O} \rightarrow p_{C1}$	28.1
		2.07 Å (C1-O)		$\pi_{C2=O} \rightarrow p_{C1}$	12.5
		102° (C1-C2=O)		$sp_{C1} \rightarrow \sigma^*_{Au-P}$	219.0
				$d_{Au} \rightarrow p_{C1}$	28.6
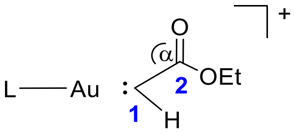 LUMO = -4.67 eV	I1a-H*	1.96 Å (Au-C1)	-0.21 (C1)	$p_{O} \rightarrow p_{C1}$	5.8
	α = 120°	2.32 Å (C1-O)		$sp^{2.7}_{C1} \rightarrow \sigma^*_{Au-P}$	270.2
		120° (C1-C2=O)		$d_{Au} \rightarrow p_{C1}$	35.2
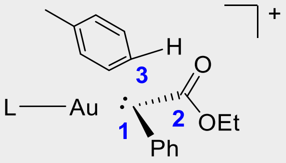	I2a-Ph-p	2.15 (Au-C1)	-0.43 (C1)	$sp^{5.0}_{C1} \rightarrow \pi^*_{C2=O}$	48.7
		1.57 (C1-C3)	0.39 (H)	$sp^{5.0}_{C1} \rightarrow \pi^*_{C=C(Ph)}$	10.9
				$\pi_{C=C(Ph)} \rightarrow sp^{3.1}_{C1}$	24.8
				$sp^{5.0}_{C1} \rightarrow \sigma^*_{Au-P}$	185.4
				$\sigma_{C3-C} \rightarrow sp^{3.1}_{C1}$	1536.0
				$\sigma_{C3-H} \rightarrow sp^{3.1}_{C1}$	19.3
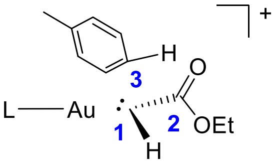	I2a-H-p	2.11 (Au-C1)	-0.65 (C1)	$sp^{4.3}_{C1} \rightarrow \pi^*_{C2=O}$	54.2
		1.55 (C1-C3)	0.37 (H)	$sp^{4.3}_{C1} \rightarrow \sigma^*_{Au-P}$	230.5
				$\sigma_{C3-C} \rightarrow sp^{2.9}_{C1}$	1589.7
				$\sigma_{C3-H} \rightarrow sp^{2.9}_{C1}$	18.1

[a]In *e*; [b]From NBO second-order perturbation analysis; [c]In kcal mol^{-1}.

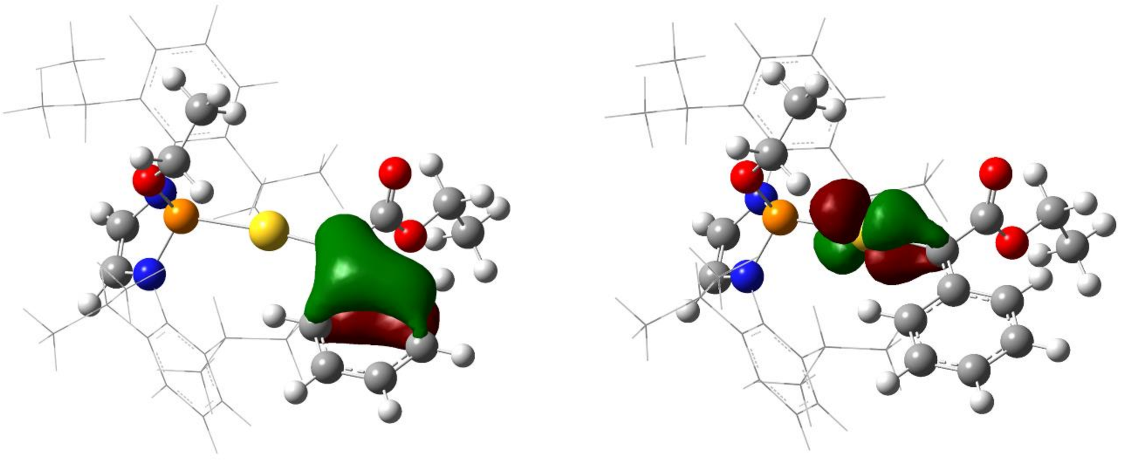

Figure S3: 3D representation for NLMOs associated with the $C{=}C_{Ph}$ π-donation to C1 (left) and π-back-donation from Au to C1 (right) for **I1a-Ph**. Isovalue: 0.05.

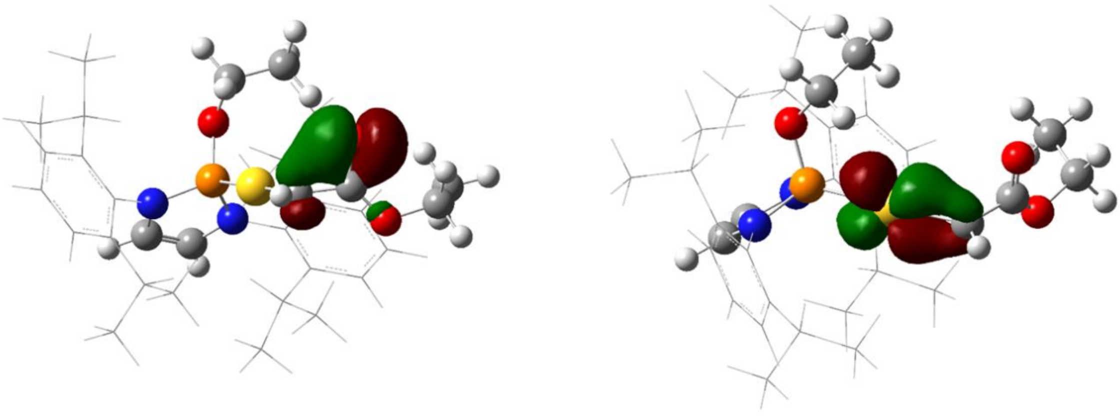

Figure S4: 3D representation for NLMOs associated with the σ-donation from O to C1 (left) and π-back-donation from Au to C1 (right) for **I1a-H**. Isovalue: 0.05.

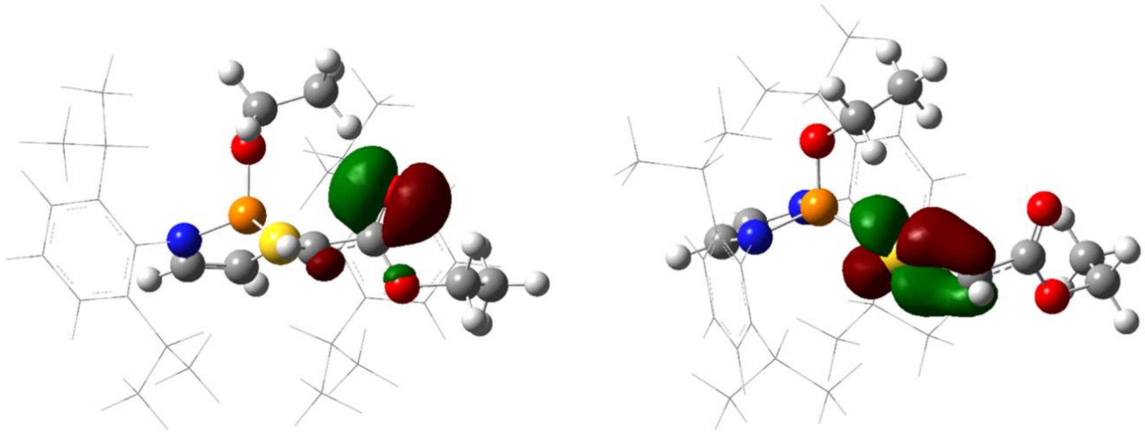

Figure S5: 3D representation for NLMOs associated with the σ-donation from O to C1 (left) and π-back-donation from Au to C1 (right) for **I1a-H***. Isovalue: 0.05.

Table S9: Selected natural charges of **I1a-Ph** and **I1a-H**.

Natural Charge	I1a-Ph	I1a-H
C1	-0.07	-0.19
Au	0.40	0.42
Carbene	0.23	0.19
ADAP ligand	0.37	0.39

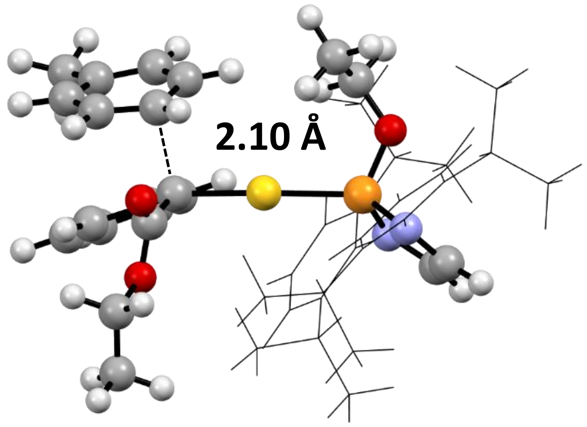

TSI1a-I2a-Ph-*p*

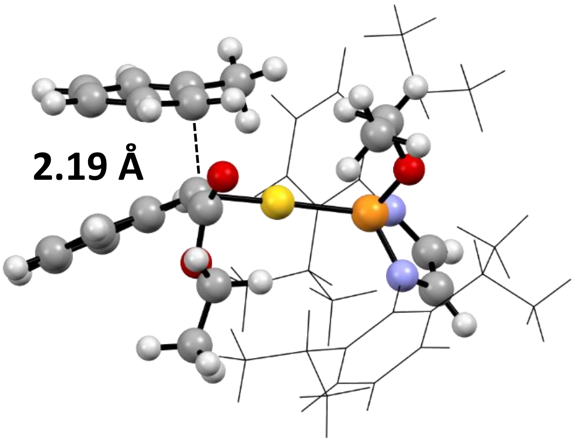

TSI1a-I2a-Ph-*o*

Figure S6: Optimized geometries for the addition TSs **TSI1a-I2a-Ph** with C-C bond formation distances yielding the *para* isomer (left) and the *ortho* isomer (right).

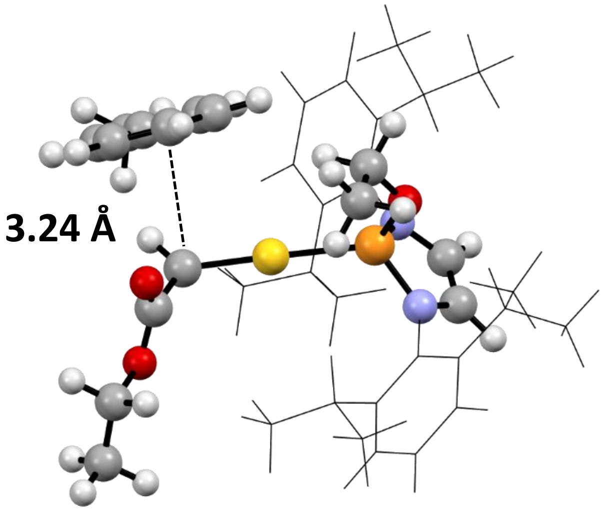

TSI1a-I2a-H-*p*

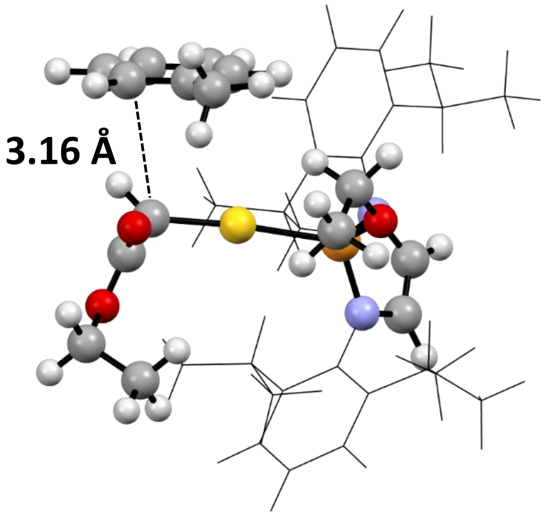

TSI1a-I2a-H-*o*

Figure S7: Optimized geometries for the addition TSs **TSI1a-I2a-H** with C-C bond formation distances yielding the *para* isomer (left) and the *ortho* isomer (right).

Table S10: Energies (G, in Hartrees) of optimised structures.

Structure	G (Hartrees)	Structure	G (Hartrees)
I1a-Ph	-2288.764839	3N-O	-2559.949499
TS-I1a-I2a-Ph-o	-2559.988700	TSN-OEt	-2559.894777
I2a-Ph-o	-2559.997466	2N-OEt	-2559.903616
I3a-Ph-o	-2560.030115	TSOEt-O-ortho-in	-2559.894121
TS-I1a-I2a-Ph-p	-2559.993780	TSOEt-O-ortho-out	-2559.890829
I2a-Ph-p	-2560.002799	2OEt-O-ortho	-2559.900301
I3a-Ph-p	-2560.044279	TS3rot-ortho	-2559.897536
I1a-H	-2057.923243	3OEt-O-ortho	-2559.938161
I1a-H*	-2057.919137	TSOEt-OEt-ortho-in	-2559.893102
TS-I1a-I2a-H-o	-2329.164105	TSOEt-OEt-ortho-out	-2559.891755
I2a-H-o	-2329.202236	2OEt-OEt-ortho	-2559.896244
I3a-H-o	-2329.247454	TS2rot-ortho	-2559.881952
TS-I1a-I2a-H-p	-2329.164921	Au	-1751.715865
I2a-H-p	-2329.205716	1NOEt-O	-2398.108204
I3a-H-p	-2329.250703	TS1N-1OEt-O	-2398.097722
toluene-methodI	-271.2517964	1NOEt-OEt	-2398.107382
1OEt-O	-2288.680884	TS1N-1OEt-OEt	-2398.098643
1OEt-OEt	-2288.681284		
TS1rot	-2288.665664		
toluene-methodII	-271.2365376		
PhEDA	-646.3729323		
TSOEt-O	-2559.896569		
2OEt-O	-2559.904067		
TS3rot	-2559.902544		
3OEt-O	-2559.950329		
TSOEt-OEt	-2559.897199		
2OEt-OEt	-2559.902833		
TS2rot	-2559.894329		
TSN-O	-2559.893881		
